# Morphometric Explanations for Deep Learning Based Neuroimaging Classifiers

**DOI:** 10.1002/hbm.70633

**Published:** 2026-08-27

**Authors:** Ankush Gajanan Arudkar, Bernard J. E. Evans, Sabrina Sghirripa, Mark Jenkinson, for the Alzheimer's Disease Neuroimaging Initiative

**Affiliations:** ^1^ Adelaide University Adelaide Australia

**Keywords:** counterfactual explanation, explainable artificial intelligence, magnetic resonance imaging

## Abstract

Despite increasing accuracy of deep‐learning‐based medical imaging classifiers for detecting complex neurodegenerative disorders, an inherent lack of transparency and interpretability in their decisions poses a challenge to their clinical and research applications. While existing explainability methods provide insights into broader aspects of a classification decision, they do not provide a direct interpretation of how specific image information was used for a classification. Geometric, or morphological, brain changes are known to exist in multiple neurodegenerative disorders, and explaining how these changes are being used by a classifier would provide domain experts with an easily interpretable understanding of morphology contributing towards the classification decision. Our work proposes a counterfactual method to explain morphological features used by deep‐learning classifiers for classifying image instances. Furthermore, to provide a more interpretable understanding of common morphological features used by a classifier for the population, we combine the instance explanations to describe average morphological brain changes used by the classifier in MNI152 space. The proposed method was tested using classifiers trained for detecting Parkinson's Disease (PD) and a simulated disease in T1‐w MRIs. The counterfactual images generated by our method were shown to explain learnt disease‐related image information, as the classification of more than 98% of images for the PD classifier was changed by modifying the explained aspects of the image morphology. By deriving geometric explanations used by deep‐learning classifiers, future research can advance trustworthiness by validating the classifiers against existing disease knowledge and potentially harness them to explore novel disease processes. Our code is available at: https://github.com/ankusharudkar/Morphometric‐Explanations.

## Highlights

- Constrained counterfactual generation improves the interpretability of generated explanations for neuroimaging classifiers.
- Geometric explanations provide a direct explanation of the anatomical morphology driving the classification process.
- Aggregating individual geometric explanations reveals common group‐level morphological changes used by the disease classifier in MNI space.

## Graphical abstract

The proposed morphometric explainability method using geometry‐constrained counterfactual generation that explains morphological changes contributing towards classification by changing the classification decision of images using morphological transforms.

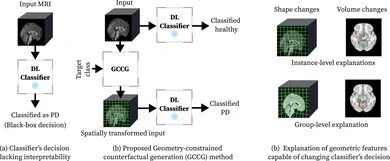

## 1 Introduction

Deep learning classifiers have been shown to detect complex neurodegenerative diseases, such as Multiple Sclerosis and Alzheimer's Disease, using structural medical imaging data with high accuracy (Basaia et al. 2019; Umirzakova et al. 2025). Nevertheless, clinical adoption of these models requires consideration of critical challenges beyond improving classification performance. Among these challenges, the lack of transparency and interpretability in model decisions have been identified as major obstacles to trusting model predictions for clinical applications (Giorgetti et al. 2025; Mienye et al. 2024; Van Der Velden et al. 2022). For diseases like Parkinson's Disease (PD), which is clinically detected by a diagnosis of exclusion as there are no in vivo diagnostic tests available (Sveinbjornsdottir 2016), deep learning classifiers based on structural brain images have achieved accuracies ranging from 75% to 95% (Camacho et al. 2023; Chakraborty et al. 2020; Hussain et al. 2025) in detecting PD. Given the high global disease burden for PD (Luo et al. 2025), these models have been proposed as a potential way to improve diagnosis and monitoring of the disease. However, their “black‐box” nature presents a challenge to trusting model decisions for patient care. To address the challenge of explainability and improve transparency in model decisions, different explainability approaches have been developed to explain aspects of a classification decision.

Feature attribution methods form a widely used class of explainability approach used for deep‐learning image classifiers. They explain a classification decision by assigning an importance score to regions of the input image, highlighting their contribution towards the classification decision. Feature attribution methods such as GradCAM (Selvaraju et al. 2017), LIME (Ribeiro et al. 2016), Integrated Gradients (Sundararajan et al. 2017), and SHAP (Lundberg and Lee 2017) have been used to explain the relative importance of brain regions for classifiers trained on neuroimaging and imaging‐derived data for PD and other neurodegenerative disorders (Alatrany et al. 2024; Camacho et al. 2024; Esan et al. 2025). For example, the hippocampus was found to be one of the most important regions used by an Alzheimer's Disease classifier. Another feature attribution method applied to a PD classifier, SmoothGrad (Smilkov et al. 2017), was used to explain which brain regions in input MRIs were important to the classifier for detecting PD (Camacho et al. 2023). However, the explanations provided by these spatial importance maps are insufficient to understand how the changes in these highlighted regions affect the classification decision. For example, whether a brain region is important due to its intensity, shape, or texture cannot be inferred directly from feature attribution explanations.

Another class of explainability methods, counterfactual methods (Roese 1997), explain why an image region was important by modifying the input image to yield different classifications. For example, by modifying a healthy MRI to produce a counterfactual image that the classifier predicts as PD, the alterations performed to the input image can be used to explain the image information used by the classifier. Counterfactual images generated by modifying the voxel (3D equivalent of a pixel) intensities in input images were used to explain clinically useful information in cardiomegaly and pleural effusion used by a chest X‐ray classifier (Singla et al. 2023) and to identify changes to midbrain voxel intensities used by an Alzheimer's disease classifier in a brain MRI (Dhinagar et al. 2024). In comparison to other explainability methods, user studies have shown that counterfactual explanations provided more clinically relevant understanding of the classification decisions (Singla et al. 2023). Although in user studies, counterfactual explanations have been shown to improve interpretability of the classification decisions, counterfactual images produced by modifying voxel intensities do not reveal the biological underpinning of these changes. For example, intensity changes in MRI could be attributed to changes in shape, tissue composition, or microstructure, limiting domain experts' ability to interpret the explanation.

To address this limitation, we propose a counterfactual explanation method that explains geometric features contributing towards classification of an image, as morphological changes are established markers for various neurodegenerative diseases (Filippi et al. 2020; Li et al. 2017; Pini et al. 2016). Our method uses topology‐preserving diffeomorphic spatial transforms to generate counterfactual images. Diffeomorphic transforms have been shown to produce high‐fidelity, anatomically plausible brain images in augmentation tasks (Pombo et al. 2023), making them suitable for generating interpretable counterfactuals. By analyzing the geometric transformations required to change a classification, our method reveals what morphological changes in brain anatomy drive the classifier's decisions.

While instance‐level explanations reveal morphological features driving individual predictions, they do not adequately capture general disease patterns. Manually examining hundreds of instance explanations is impractical and may introduce selection bias. Therefore, we aggregate spatial transformations across all instances in MNI152 space (Grabner et al. 2006) (a standard brain reference space allowing voxel‐wise comparisons across subjects) to identify common morphological features associated with disease classification. This produces a group‐level explanation of the anatomical changes the classifier has learnt to associate with the disease. Based on the widely accepted taxonomy outlined in Guidotti et al. (2019), our method provides example‐based, model‐specific explanations for differentiable image classifiers, offering both local (instance‐level) and global (group‐level) insights into the underlying image morphology. We validate this approach on MRI classifiers trained on a PD dataset and a simulated dataset with known disease features.

Our contributions, therefore, are as follows:

- We propose a geometry‐constrained counterfactual generation method for explaining morphological changes used by structural imaging classifiers to detect disease in image instances.
- We provide a group‐level explanation of common morphological changes in MNI152 space used by the classifiers to detect the disease in images.

## 2 Methods

### 2.1 PD Dataset

To train the PD classifier, data were sourced from three different studies consisting of PD patients and healthy controls. The studies included were: the Parkinson's Progression Markers Initiative (PPMI), (Marek et al. 2018); the Alzheimer's Disease Neuroimaging Initiative (ADNI), (Petersen et al. 2010) database; and the Open Access Series of Imaging Studies‐1 (OASIS‐1), (Marcus et al. 2007). While the PD patients were obtained from PPMI, the other two studies were included to augment the number of healthy controls in the data. PD patients and healthy controls who had whole‐brain T1‐weighted MRIs were included in the combined dataset.

The consolidated PD dataset consisted of 3841 images from 1289 subjects: 459 patients with PD and 830 healthy controls (CN), as detailed in Table 1. To ensure data consistency across different models trained on the dataset, subjects, and their respective images were partitioned into a single training, validation, and testing split. The training dataset, consisting of 624 sex‐ and age‐matched participants with ±2 years tolerance (312 PD and 312 CN subjects), was used to train the models. Similarly, a separate set of 54 participants (27 PD and 27 healthy) was used for testing the models, while the remaining 611 participants were assigned to the validation dataset. Due to an imbalance in participants demographics, we ended up with higher number of unbalanced participants in the validation dataset after picking a balanced set for the training and the testing datasets. An overview of resulting cohort composition for training and testing splits is shown in Table 2.

**TABLE 1:** Overview of Parkinson's Disease (PD) and control (CN) subjects included in the study.

Dataset	Group	Subjects	Sex (% female)	Mean age in years (SD)	Image count
PPMI	PD	459	39.8	64.5 (10.9)	1184
	CN	142	34.5	61.4 (9.3)	242
ADNI	CN	372	51.1	74.4 (5.9)	2079
OASIS‐1	CN	316	62.3	45.1 (23.9)	336

*Note:* The subjects were sourced from the Parkinson's Progression Markers Initiative (PPMI; Marek et al. 2018), the Alzheimer's Disease Neuroimaging Initiative (ADNI; Petersen et al. 2010), and the Open Access Series of Imaging Studies‐1 (OASIS‐1; Marcus et al. 2007).

**TABLE 2:** The cohort composition of the participants in training and testing datasets.

Split	${\text{PPMI}}_{\mathrm{PD}}$	${\text{PPMI}}_{\mathrm{CN}}$	${\text{ADNI}}_{\mathrm{CN}}$	${\text{OASIS}}_{\mathrm{CN}}$
Train	50%	8%	19%	21%
Test	50%	27%	3%	18%

### 2.2 Simulated Disease Dataset

**FIGURE 1:**
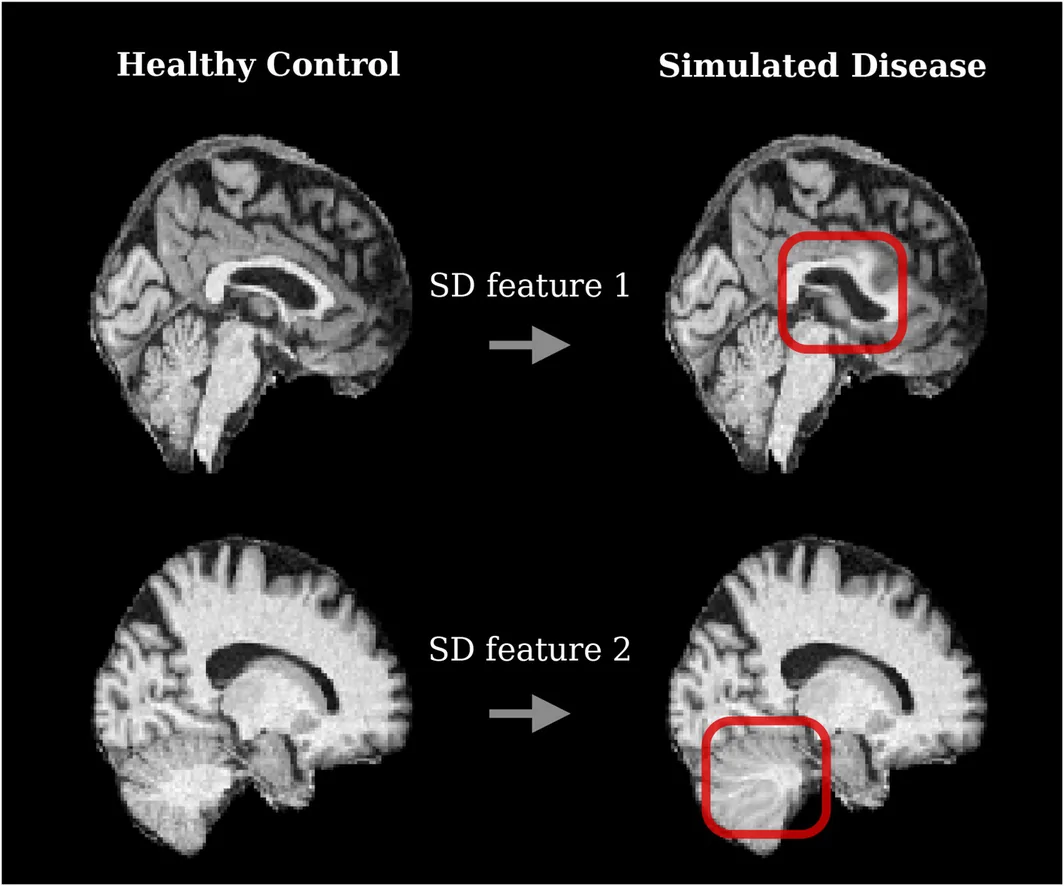
Example slices of the simulated disease (SD) features used to generate the simulated dataset. The bounding boxes indicate the locations where a pre‐defined 3D Gaussian‐based spatial transformation was applied to an image to generate the corresponding SD image.

We created a simulated disease (SD) dataset with known geometric changes to validate our method. To generate the SD images, we introduced one of two artificial disease features into the preprocessed MRI images (described in Section 2.3). The two features simulate heterogeneity in disease manifestation, which is common in many neurological disorders. We chose the features to be: (1) atrophy of white matter in the cerebellum, and (2) deformation in the right ventricle, as shown in Figure 1. Smooth Gaussian deformation templates, created separately for both features, were applied at fixed coordinate locations to the preprocessed MRIs to create the SD images. The details of these simulated features can be found in Appendix A.

The SD dataset used the same training, validation, and testing participants as the PD dataset. To generate the dataset, each participant was randomly assigned to either the SD or CN group with equal probability. Then, for each image of the SD participants, one of the two mentioned disease features was randomly introduced, with 50% probability for each feature, in the image to generate the simulated disease MRI. By using this set of generated images and data split, data consistency was ensured across the classification and counterfactual SD models.

### 2.3 Data Preprocessing

Acquired T1‐weighted MRIs were preprocessed by following a sequence of four steps to standardize the image orientations, intensities, and voxel sizes to make them suitable for deep‐learning models. First, to remove non‐brain tissue from the MRIs, brain extraction was performed using SynthStrip (Hoopes et al. 2022). Next, the brain extracted images were corrected for bias fields using FSL's FAST tool (Zhang et al. 2001) to remove radio‐frequency field inhomogeneities. Subsequently, the images were affine registered with 12 degrees‐of‐freedom to the 1 mm T1‐weighted MNI152 template provided in FSL, using FSL's FLIRT tool (Jenkinson et al. 2002, 2012; Jenkinson and Smith 2001), resulting in an image of size 182×218×182 voxels. Affine registration was used to avoid non‐linear geometric distortions in anatomy. Finally, due to the higher computational cost involved in training larger image sizes, the affine registered images were down‐sampled to 128×128×128 voxels with trilinear interpolation to train the deep learning models, effectively changing the voxel dimensions to 1.41×1.70×1.41 mm.

### 2.4 Classification Models

**FIGURE 2:**
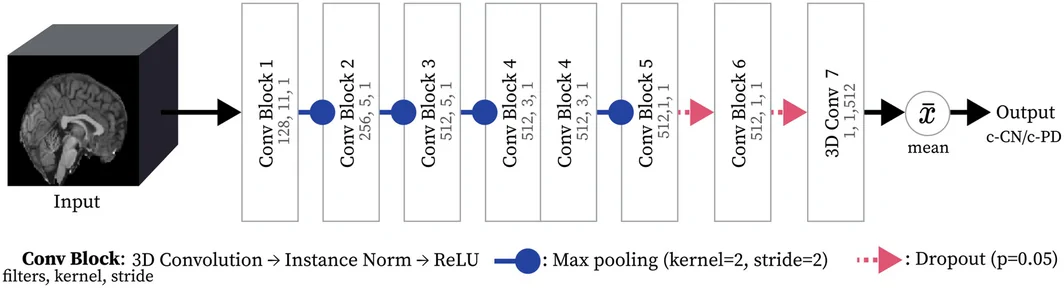
The fully convolution architecture used for the deep‐learning classification models trained on the Parkinson's Disease and the simulated disease datasets.

We trained a PD classifier ($\text{Classifie}r_{\mathrm{PD}}$) due to lack of publicly available, pre‐trained models. A fully convolutional architecture was adopted due to its prevalence in deep‐learning classifiers trained on 3D volumetric medical imaging. This classifier consisted of blocks of 3D convolutional layers (Tran et al. 2014), instance normalization (Ulyanov et al. 2016), and ReLU (Agarap 2018) activation function as shown in Figure 2. Additionally, element‐wise dropout regularization was applied to the inputs of the final two layers of the network to prevent over‐fitting, with 0.05 dropout probability. The classifier was optimized to minimize binary cross‐entropy loss (BCE) on the training data. The same architecture was also used for the SD classifier ($\text{Classifie}r_{\mathrm{SD}}$) on the simulated disease training dataset.

To differentiate the real labels from the labels predicted by the models, we prefix the classified labels with “c‐.” This distinction clarifies the labelling of the counterfactual images by the classifier, especially as real labels cannot be obtained for the synthesized counterfactual images.

#### 2.4.1 Classifier Training Details

Both the classifiers were trained using the Adam optimizer (Kingma and Ba 2014) with hyper‐parameter settings of: $\beta_{1}=0.9$, $\beta_{2}=0.999$, a learning rate of $10^{-5}$, and a batch size of 8 due to computational limitations. These training hyper‐parameters were empirically chosen for both PD and SD datasets. Furthermore, to prevent over‐fitting, four image augmentations were randomly applied, each with 50% probability, to the training images: (1) random global translations (±5 voxels), (2) random global rotations ($\pm 20^{{}^{\circ}}$), (3) random bias field as implemented in Cardoso et al. (2022) with three degrees of freedom and coefficients randomly ranging between 0 and 0.1, and (4) random occlusions of image sub‐volumes with a maximum side length of 40 voxels. The global, linear transformations were chosen to preserve the morphology of anatomical structures while still retaining approximate orientation of the images in the field of view. Whereas, the bias field and occlusion were included to reduce overfitting to residual radio‐frequency inhomogeneities and promoting classification features to be learnt from the entire image.

### 2.5 Counterfactual Image Properties

For the counterfactual images to provide meaningful explanations, three properties need to be satisfied (Bayrak and Bach 2024): validity, fidelity, and proximity.

#### 2.5.1 Validity

A valid counterfactual transform converts an image so that the label given to this counterfactual image by the classifier is different from the label given to the original image by the classifier. This is a basic requirement for the counterfactual transform to represent changes that are actually used for classification. Validity was measured by classification conversion rates, defined as the proportion of images converted from class A to class B for every combination of the original source and the predicted destination classes.

#### 2.5.2 Fidelity

To obtain meaningful explanations, the counterfactual changes applied to images should result in realistic images that maintain biological plausibility as represented by the distribution of training images. In the absence of human evaluators, we applied the following widely used metrics to quantify fidelity of the generative images: Fréchet Inception Distance (FID) (Heusel et al. 2017), Mean Maximum Discrepancy (MMD) (Gretton et al. 2012), and Multi‐scale Structural Similarity Index Measure (MS‐SSIM) (Wang et al. 2003). The metrics were computed for the set of real and corresponding counterfactual images using the PyTorch *ignite* library. While MMD and MS‐SSIM scores are evaluated directly using the sets of images, an intermediary image feature‐extractor is used by FID. This makes the scale of the FID scores dependent on the feature extractor being used. Following Sun et al. (2022), we used a 3D‐ResNet50 pre‐trained on RadImageNet as our feature extractor to maintain consistency with the other methods to which we compare our results.

Additionally, for geometry‐constrained counterfactual transforms, we compute Jacobians of the counterfactual displacement fields to ensure topology is preserved by the transform. The values of the Jacobian determinant indicate the ratio of volumes before and after change for a voxel. By ensuring the volume ratio is always greater than 0, we validate that the spatial transformation is topology‐preserving and invertible, which supports its biological plausibility. This property, however, could not be validated for the unconstrained counterfactual transforms.

#### 2.5.3 Proximity

Proximity ensures that the counterfactual changes are minimal, as smaller, local modifications are more easily interpreted than whole‐brain alterations. It was measured by computing the average, across a set of images, of the mean absolute intensity differences between input and corresponding counterfactual images.

### 2.6 Geometry‐Constrained Counterfactual Generation

To generate the geometry‐constrained counterfactual images for explaining a classifier, we trained a generative model to predict spatial deformations for transforming the input images. We adopted a Generative Adversarial Network (GAN) (Goodfellow et al. 2014) as the generative model since its variants have been shown to generate high‐fidelity 3D volumetric T1‐w MRIs (Sun et al. 2022; Tudosiu et al. 2024). Moreover, GANs have also been used to successfully generate unconstrained counterfactual images for structural medical images (Dhinagar et al. 2024; Singla et al. 2023).

**FIGURE 3:**
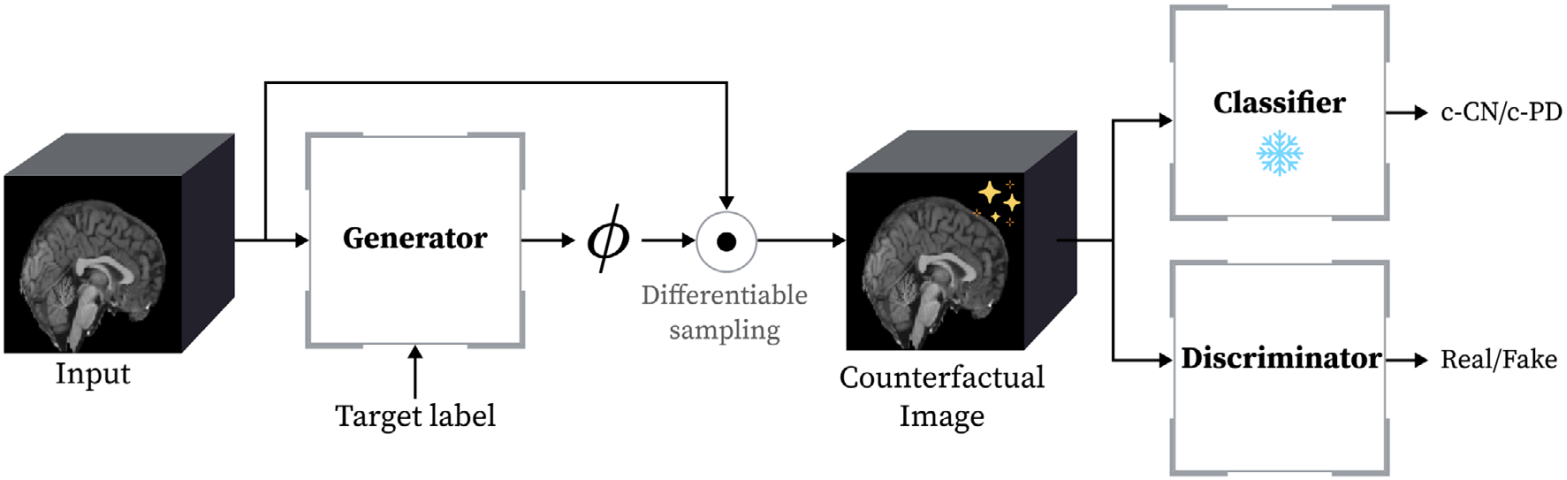
An overview of the proposed Geometry‐Constrained Counterfactual Generation (GCCG) method. A conditional generator produces a counterfactual transform (displacement field, ϕ) that, when applied to the input image, aims to change the prediction of a pre‐trained deep‐learning classifier to a given target label.

The proposed Geometry‐Constrained Counterfactual Generation (GCCG) method uses a conditional generative process based on StarGAN (Choi et al. 2018) to produce a counterfactual image for a given pair of an input image and the desired target label. The conditional generation provides flexibility of deciding the target label which would be essential for classifiers with more than two classes for prediction. To generate the counterfactual image, a displacement field predicted by the generative model is applied to the input image. The generative process was trained to produce valid counterfactual images for a pre‐trained classifier while also promoting fidelity and proximity of the transforms as described in Section 2.6.1. An overview of the proposed geometry‐constrained counterfactual generation process is shown in Figure 3.

#### 2.6.1 Generator

**FIGURE 4:**
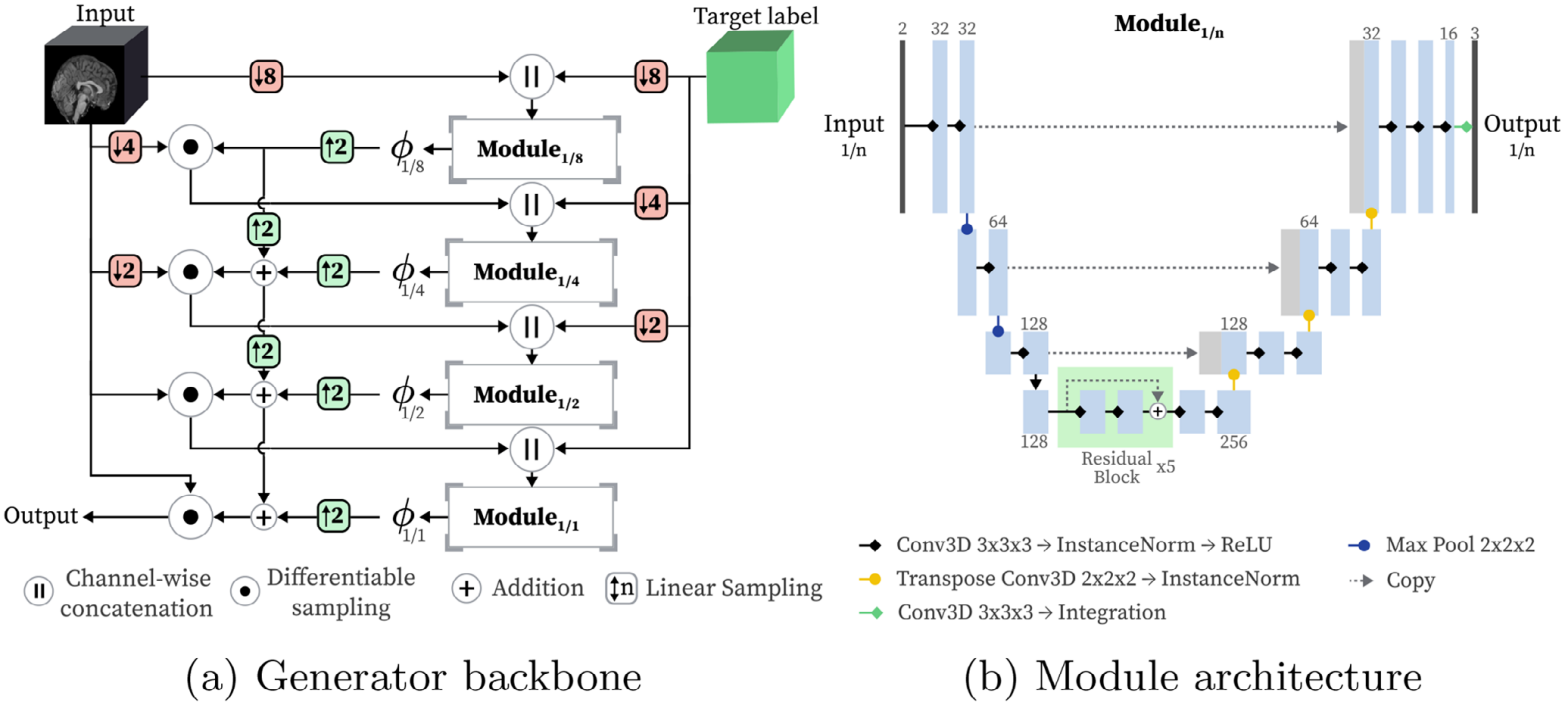
Generator architecture for the counterfactual generation process, where (a) shows the hierarchical generation architecture and (b) shows the architecture of the modules used in the hierarchical model.

The architecture of the generator (G) for the proposed counterfactual generation model used a multi‐step prediction process adapted from Strittmatter and Zöllner (2024). This architecture, as shown in Figure 4a, predicts the counterfactual spatial transformation for the input image by refining the spatial transform in multiple steps with increasing resolutions. It was shown to outperform multiple state‐of‐the‐art deep learning approaches for deformable image registration and hence was adopted for the generator. Moreover, each step within the generator used a U‐Net (Ronneberger et al. 2015) architecture with residual bottleneck layers (He et al. 2015) as shown in Figure 4b.

The U‐Net within each module in the generator produces a velocity field (v) which is integrated using the scaling and squaring method, as described in Balakrishnan et al. (2019), to output a diffeomorphic displacement field (ϕ). The diffeomorphic property ensures that the spatial transformation is invertible and preserves image topology, which is necessary for the biological plausibility of the counterfactual spatial transforms. The displacement field output by the final module is applied to the input image using a differentiable image sampling method, as described in Jaderberg et al. (2015), to generate the counterfactual output image.

We represent the generator G as a function of an input image x and a target label t which produces the counterfactual image $G\left(x,t\right)$ as its output. The generator was trained by using a combination of loss functions and regularization terms as described next.

*Classification loss* ($L_{C}$) is optimised to encourage the generator to make valid counterfactual images for the classifier (C) as specified by the target labels. For an input image x and its desired target label t, the classification loss was computed between the predicted label for the counterfactual image $C\left(G\left(x,t\right)\right)$ and the target label as described in Equation (1).

(1) $$L_{C}=L_{\mathrm{BCE}}\left(C\left(G\left(x,t\right)\right),t\right).$$

*Adversarial loss* ($L_{\mathrm{Adv}}$) incorporates feedback from the discriminator in the counterfactual generation process to make the distribution of generated images similar to the distribution of training images. The patch loss formulation adapted from Isola et al. (2017) was used to minimize mean BCE loss between $N_{d}=4\times 4\times 4$ patch labels, as predicted by the discriminator (1:real or 0:fake) for the counterfactual images, and the real patch labels for the images as described in Equation (2), where i,j,k represent indices for elements in 3D volume output by the network D.

(2) $$L_{\mathrm{Adv}}=\frac{1}{N_{d}}\sum_{i,j,k}L_{\mathrm{BCE}}\left(D{\left(G\left(x,t\right)\right)}_{i,j,k},1\right).$$

*Smoothing regularization* ($L_{\text{smooth}}$) was used to avoid sharp deformations in the predicted displacement fields, similar to the regularization used in Balakrishnan et al. (2019). It was computed for the displacement fields by using the difference between the neighboring displacement vectors for each voxel as described in Equation (3).

(3) $$L_{\text{smooth}}=\sum_{i,j,k}{\left\|\nabla \phi_{i,j,k}\right\|}^{2}.$$

*Displacement field norm* ($L_{\mathrm{norm}}$) was used as another regularization term to promote proximity between the input and counterfactual images. It was computed as the total $L^{2}$ norm of the displacement vectors to penalise larger displacements in the predicted displacement fields as shown in Equation (4).

(4) $$L_{\mathrm{norm}}=\sum_{i,j,k}{\left\|\phi_{i,j,k}\right\|}_{2}.$$

*Global structure preservation* ($L_{\text{blur}}$) was introduced as a regularization term that aimed to reduce the disparity between broader structural features of the input and the generated counterfactual images. It was computed as the mean squared error between blurred versions of the input and generated images. The blur was applied by convolving a 5×5×5 averaging filter ($F_{5}$) over the images as described in Equation (5) where “⊗” represents the convolution operation and $N_{v}$ is the number of voxels in the images. The size of the blurring filter can be adjusted according to the magnitude of voxel displacements being considered in the study; it was chosen to blur regions of 5×5×5 voxels to penalise larger‐scale deformations while allowing for local deformations.

(5) $$L_{\text{blur}}=\sum_{x}\frac{1}{N_{v}}{\left(F_{5}⊗x-F_{5}⊗G\left(x,t\right)\right)}^{2}.$$

Using the above mentioned terms, the generator loss $L_{G}$ was computed as a linear combination of all terms along with a multiplicative term, as shown in Equation (6). This multiplicative term was found to improve training stability as presented in Appendix B.

(6) $$\begin{array}{c} L_{G}=\alpha_{1}L_{C}+\alpha_{2}L_{\mathrm{Adv}}+\alpha_{3}L_{\text{smooth}}+\alpha_{4}L_{\mathrm{norm}}+\alpha_{5}L_{\text{blur}}\\ \;+L_{C}L_{\mathrm{Adv}}L_{\text{smooth}}L_{\mathrm{norm}}L_{\text{blur}}. \end{array}$$

#### 2.6.2 Discriminator

**FIGURE 5:**
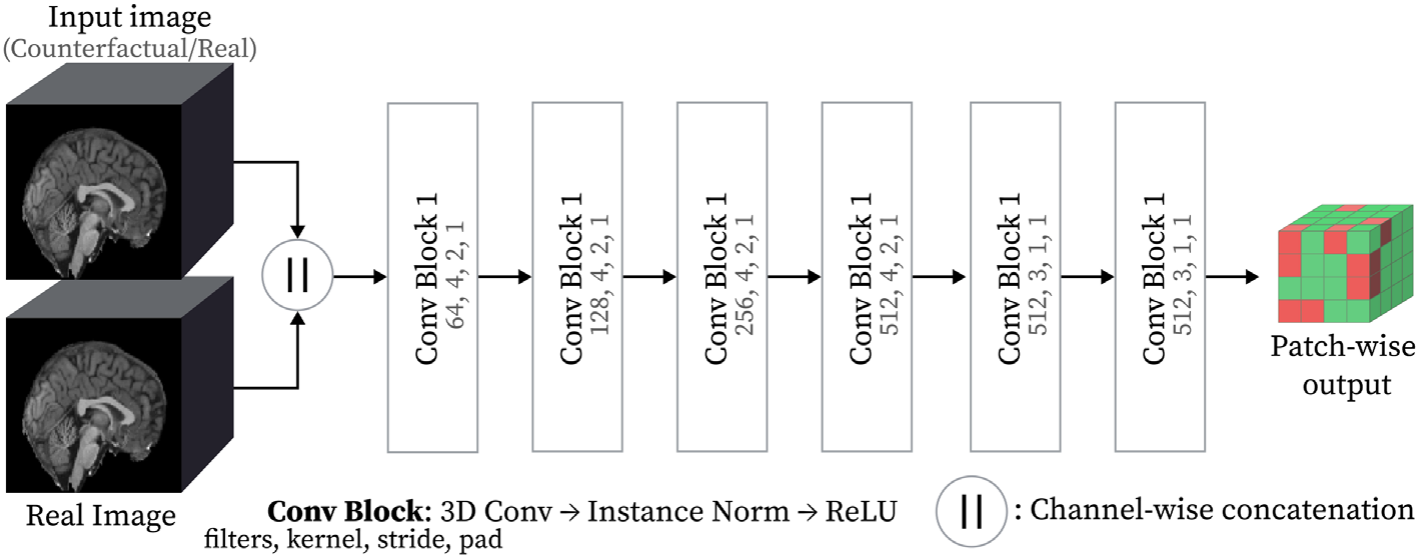
Patch discrimination architecture, adapted from PatchGAN (Isola et al. 2017), used as discriminator in the proposed counterfactual generation model.

The discriminator (D) for the generative counterfactual model was adapted from the architecture proposed by Isola et al. (2017) for PatchGAN. Unlike traditional discriminator architectures that assign a real or fake label to the entire input image, a patch discriminator predicts a real or a fake label for patches within the input image. This approach was shown to improve training stability and generation fidelity in GANs and hence was adopted for the discriminator architecture, as shown in Figure 5.

The input to the discriminator was a channel‐wise concatenation of a real image and either a generated or real image, and it produced a $N_{d}=4\times 4\times 4$ volume. Each element in the predicted output volume provides a similarity score for corresponding patches in the pair of input images. The discriminator was optimised to minimise the loss ($L_{\text{Disc}}$), defined as the sum of binary cross entropy losses for patches from the pair of images and real patch labels, as shown in Equation (7).

(7) $$L_{\text{Disc}}=\frac{1}{N_{d}}\left(\sum_{i,j,k}L_{\mathrm{BCE}}\left(D{\left(x\right)}_{i,j,k},1\right)+\sum_{i,j,k}L_{\mathrm{BCE}}\left(D{\left(G\left(x\right)\right)}_{i,j,k},0\right)\right).$$

#### 2.6.3 Training Details

The geometry‐constrained counterfactual generation models were trained using the Adam optimizer (Kingma and Ba 2014) with empirically set hyper‐parameter values of: $\beta_{1}=0.9,\beta_{2}=0.999$, a learning rate of $10^{-4}$, and a batch size of 2 due to computational limitations. The training was split across two NVIDIA‐A100 GPUs using a data distributed parallel strategy as implemented in PyTorch. The models were trained for a maximum of 200 epochs with early stopping criterion applied when the total validation loss did not reduce for 15 consecutive steps. While training, image augmentations of global translations (±5 voxels) and global rotations of (±10 degrees) were applied to images. The values of loss term coefficients in the generator loss were empirically chosen to be: $\alpha_{1}=1$, $\alpha_{2}=1$, $\alpha_{3}=500$, $\alpha_{4}=10^{-4}$, $\alpha_{5}=1$.

### 2.7 Unconstrained Counterfactual Generation

To compare the explanations produced by the proposed method to existing counterfactual methods, we compared the proposed GCCG method to counterfactual methods where no systematic constraint was applied to the generation process (Dhinagar et al. 2024; Singla et al. 2023). These methods, here referred to as unconstrained counterfactual generation (UCG) methods, modify the voxel intensities of the input image to produce the corresponding counterfactual image. The GCCG architecture was modified to train the UCG models by performing two main changes to the model architecture and training process: (1) the generator predicts an additive field instead of a displacement field, which is applied to the input image intensities by performing matrix addition to generate the unconstrained counterfactual image, (2) the displacement field smoothing ($L_{\text{smooth}}$) and global structure preservation ($L_{\text{blur}}$) were dropped from the generator loss while the displacement field norm ($L_{\mathrm{norm}}$) was replaced by norm of additive field, to make the formulation comparable to other mentioned unconstrained counterfactual generation methods.

#### 2.7.1 Training Details

The UCG models for the PD and the SD datasets were trained with similar device configurations and image augmentations as the GCCG models (as outlined in Section 2.6.3). The loss term coefficients for the generator were empirically chosen to be: $\alpha_{1}=1$, $\alpha_{2}=1$, and $10^{-3}$ as the coefficient for additive norm term. The models were trained for a maximum of 200 epochs with early stopping applied when the total validation loss term did not reduce for 15 consecutive steps.

### 2.8 Instance Explanations

An instance explanation describes aspects in an image that contributed towards its classification. Depending on the image transformation, additive or displacement, we measured intensity, geometric, or volumetric changes between the original and the counterfactual images.

#### 2.8.1 Intensity Changes

The voxel‐wise intensity differences between the input and the counterfactual images, a common measure of image differences, were computed to highlight the regions modified by the transforms. They were computed for both the unconstrained and the proposed geometry‐constrained methods to analyse altered regions.

#### 2.8.2 Geometric Changes

For the proposed GCCG method, the shape changes between the input and the counterfactual images can be obtained from the predicted spatial deformation fields. Since geometric explanations cannot be accurately isolated from the additive intensity maps predicted by the UCG method, they were only computed for the GCCG models.

#### 2.8.3 Volumetric Changes

Voxel‐wise volume changes were computed for geometry‐constrained counterfactual images to provide an explanation for regional expansion and contraction in brain regions associated with the prediction of disease by the classifier. They were obtained by computing Jacobian determinants of the counterfactual displacement fields using the SimpleITK library (Lowekamp et al. 2013). The voxels with determinants equal to one indicate no change in their volume, whereas values greater than or less than one indicate an increase or decrease in voxel volumes, respectively. Furthermore, determinant values less than or equal to zero indicate biologically implausible deformations performed by folding the regional topology. This property was used to verify the preservation of topology by the counterfactual displacement fields.

### 2.9 Necessity and Sufficiency of Local Counterfactual Changes

**FIGURE 6:**
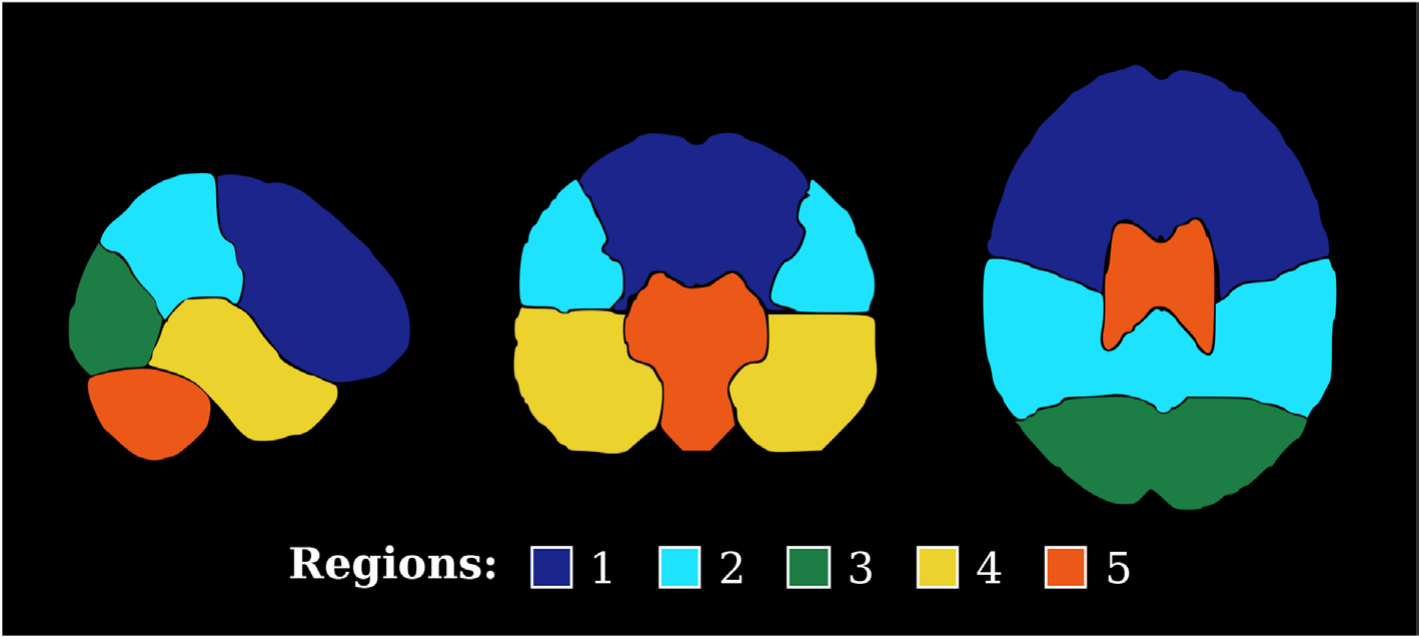
The defined regions of interest used to evaluate localized necessity and sufficiency of the predicted counterfactual transforms (adapted from Toro et al. (2009)).

The saliency of predicted counterfactual changes applied to an anatomical region does not directly establish their causal role in changing the classification decision. To further validate the fidelity of the produced morphometric explanations, we evaluated the necessity and sufficiency of the counterfactual deformations across different brain regions. These regions were defined by dividing the brain into five regions as defined by Toro et al. (2009), combining the left and right lateral regions as shown in Figure 6.

The necessity of the deformations predicted for a region was quantified by evaluating the average classification conversion rate when the deformations from that specific region were removed. Conversely, the sufficiency of a region was evaluated by preserving the predicted counterfactual changes only within the region of interest while removing them elsewhere.

### 2.10 Counterfactual Transform Strength Plot

We examined the effect of the transform strength of the predicted transforms on classification probability to analyze their sensitivity and stability. For a stable counterfactual transform, we would expect to see a smooth increase in the prediction probability as the applied transform strength (scaling factor) increases from 0 (same as the input image with no transform applied) to 1 (predicted transform). This stability in the transform can then explain the stability in the classifier's modelled relationship between the learnt disease features and disease probability.

The sensitivity of the counterfactual transforms was visually examined using a series of box plots showing the distribution of the classification probabilities for counterfactual images generated by varying strengths of the transforms. The strength of the predicted transform was varied by multiplying it by values between ±1. This range was chosen to preserve the diffeomorphic property of the predicted spatial transformations, which is not guaranteed outside this range by the generation process. The same range for the varied strength was also chosen for additive transforms, to limit the voxel intensity range of counterfactual images within the normalized intensity range, as described in Section 2.3.

### 2.11 Average Explanations

Average transforms enable an explanation of what the classifier is typically doing without having to present multiple individual examples to a user and have them figure out the common patterns themselves. These average counterfactual transforms were computed in MNI152 space, a well recognized anatomical brain space, to identify the general disease patterns learnt by the classifier. The counterfactual transforms were registered to MNI152 space using registration fields predicted by a deformable deep‐learning image registration network adapted from Balakrishnan et al. (2019) and Strittmatter and Zöllner (2024). The deformation fields predicted by the registration network were directly applied to scalar additive fields produced by UCG models to map them to MNI152 space. However, this process of scalar registration cannot be simply applied to register the vector displacement fields. We detail an algorithm to register the predicted discrete displacement fields to MNI152 space using a registration field in Appendix D. Using the same method, the validity, fidelity, and proximity metrics were computed for the average transforms, after mapping them back to native image space.

### 2.12 Integrated Gradients Explanation

Feature attributions form a prevalent class of explainability methods used for medical imaging classifiers. The explanations produced using Integrated Gradients (Sundararajan et al. 2017), a feature attribution explainability method, were generated to compare the strengths and weaknesses of the explanations produced by feature attribution and counterfactual explanation methods. The average of registered feature attributions maps were produced for correctly classified images, separately for instances classified by $\text{Classifie}r_{\mathrm{PD}}$ and $\text{Classifie}r_{\mathrm{SD}}$.

## 3 Results

### 3.1 Classification Performance

The prediction results, evaluated using a range of metrics, for the classifiers trained on the PD training dataset ($\text{Classifie}r_{\mathrm{PD}}$) and the SD training dataset ($\text{Classifie}r_{\mathrm{SD}}$) are shown in Table 3. The models were evaluated using accuracy, precision, true positive rate, false positive rate, and ROC‐AUC metrics. The $\text{Classifie}r_{\mathrm{PD}}$ achieved an accuracy of 77.9% with a bootstrapped 95% confidence interval (72.3%, 83.1%) on the testing PD dataset with 1000 iterations, which is comparable to Camacho et al. (2023). Additionally, the classifier trained on the simulated dataset achieved an accuracy of 99.8% on the SD testing dataset.

**TABLE 3:** Classification model performance measured using the following metrics: Accuracy (ACC), precision (PRE), true positive rate (TPR), false positive rate (FPR), and ROC‐AUC (AUC).

Model	Dataset	ACC	PRE	TPR	FPR	AUC
$\text{Classifie}r_{\mathrm{PD}}$	Test PD	77.9	64.3	87.1	27.2	86.7
$\text{Classifie}r_{\mathrm{SD}}$	Test SD	99.8	99.5	100	0.5	99.9

### 3.2 Counterfactual Image Properties

For the geometry‐constrained and the unconstrained counterfactual generation models, we measured their validity, fidelity, and proximity. The GCCG models trained for $\text{Classifie}r_{\mathrm{PD}}$ and $\text{Classifie}r_{\mathrm{SD}}$ are referred as $\mathrm{GCC}G_{\mathrm{PD}}$ and $\mathrm{GCC}G_{\mathrm{SD}}$ respectively. Similarly, $\mathrm{UC}G_{\mathrm{PD}}$ and $\mathrm{UC}G_{\mathrm{SD}}$ denote unconstrained counterfactual generation models for PD and SD classifiers.

#### 3.2.1 Validity

Both counterfactual generation methods achieved conversion rates greater than 98% for the PD and SD models as shown in Table 4.

**TABLE 4:** Classification conversion rates for counterfactual images generated by unconstrained (UCG) and geometry‐constrained (GCCG) counterfactual generation models for the PD and SD test datasets.

			Target class	
Dataset	Model	Source class	c‐CN	c‐PD/c‐SD
Test PD	$\mathrm{GCC}G_{\mathrm{PD}}$	CN	100	100
		PD	100	100
	$\mathrm{UC}G_{\mathrm{PD}}$	CN	100	98.5
		PD	100	100
Test SD	$\mathrm{GCC}G_{\mathrm{SD}}$	CN	99.6	100
		SD	100	100
	$\mathrm{UC}G_{\mathrm{SD}}$	CN	99.6	100
		SD	100	100

#### 3.2.2 Fidelity

The fidelity metrics computed for the images generated by the proposed GCCG and the UCG methods were found to be relatively similar, as seen in Table 5. The MMD values for both methods were computed to be 0 by the PyTorch *Ignite* library due to numerical precision limitations. Additionally, we also present metrics reported by other published methods that generated whole brain 128×128×128 voxels T1‐w MRIs, which were evaluated on the datasets used in their respective studies.

**TABLE 5:** Quantitative image fidelity metrics reported in related works and, separately, evaluated on the testing PD dataset for our own approach for geometry‐constrained counterfactual generation ($\mathrm{GCC}G_{\mathrm{PD}}$) and unconstrained counterfactual generation ($\mathrm{UC}G_{\mathrm{PD}}$) models as tested on the PD test dataset.

Method	MS‐SSIM↑	MMD↓	FID↓
HA‐GAN (Sun et al. 2022)	0.66	1.4e‐6	0.0047
LDM (Pinaya et al. 2022)	0.65	—	0.0076
VQ‐VAE (Tudosiu et al. 2024)	0.67	9.5e‐7	0.0026
$\mathrm{GCC}G_{\mathrm{PD}}$ (Ours)	0.80	**0**	**0.0007**
$\mathrm{UC}G_{\mathrm{PD}}$ (Ours)	**0.81**	**0**	0.0013

*Note:* Missing data is shown as —. The values highlighted in bold represent the best performing metrics in each column.

#### 3.2.3 Proximity

The average of absolute mean intensity difference between the input image and generated counterfactual instances was found to be less than 0.02 for both counterfactual generation methods. Since the image intensities were normalized during data preprocessing steps, this change in intensity represents less than 2% change in average voxel intensity for generating counterfactual images, as presented in Table 6. The average proximity between two randomly chosen images that were registered to the MNI152 template using the trained registration network was found to be 0.02, demonstrating that the counterfactual proximity was, on average, similar to that of two random MNI152 registered MRIs.

**TABLE 6:** Average of mean absolute intensity difference between voxels of generated counterfactual and input image instances for geometry‐constrained ($\mathrm{GCC}G_{\mathrm{PD}}$) and unconstrained ($\mathrm{UC}G_{\mathrm{PD}}$) counterfactual generation methods applied to $\text{Classifie}r_{\mathrm{PD}}$ as measured on the PD test dataset.

Model	Mean intensity distance	
	PD to c‐CN	CN to c‐PD
$\mathrm{GCC}G_{\mathrm{PD}}$	1.01e‐2	9.4e‐3
$\mathrm{UC}G_{\mathrm{PD}}$	6.4e‐4	6.3e‐4

### 3.3 Necessity and Sufficiency of Local Counterfactual Deformations

For the $\text{Classifie}r_{\mathrm{PD}}$, deformations predicted by $\mathrm{GCC}G_{\mathrm{PD}}$ for no single region were found to be necessary for converting the classification decision, as shown by the low conversion rates for necessity of regions in Table 7. In contrast, deformations for multiple regions exhibited high sufficiency in converting the image classifications. Notably, deformations restricted entirely to Region 5 (containing the midbrain) were sufficient to flip the classification for > 90% of both PD and CN images.

**TABLE 7:** Necessity and sufficiency of brain regions in converting classification of $\text{Classifie}r_{\mathrm{PD}}$ by counterfactual deformations generated by $\mathrm{GCC}G_{\mathrm{PD}}$.

ROI	Necessity (%)		Sufficiency (%)	
	CN to c‐PD	PD to c‐CN	CN to c‐PD	PD to c‐CN
1	0.0	0.0	3.15	14.1
2	0.0	0.0	20.0	57.8
3	0.0	0.0	8.9	53.12
4	0.0	1.6	52.6	78.9
5	0.0	4.2	93.2	99.2

Similar analysis for $\text{Classifie}r_{\mathrm{SD}}$ revealed only Region 5, where the simulated disease features were introduced, to be necessary and sufficient for converting classification of more than 99% of the images. By comparison, deformations in no other region were found to be necessary or sufficient in changing the decisions of $\text{Classifie}r_{\mathrm{SD}}$.

### 3.4 Counterfactual Transform Strength Plot

**FIGURE 7:**
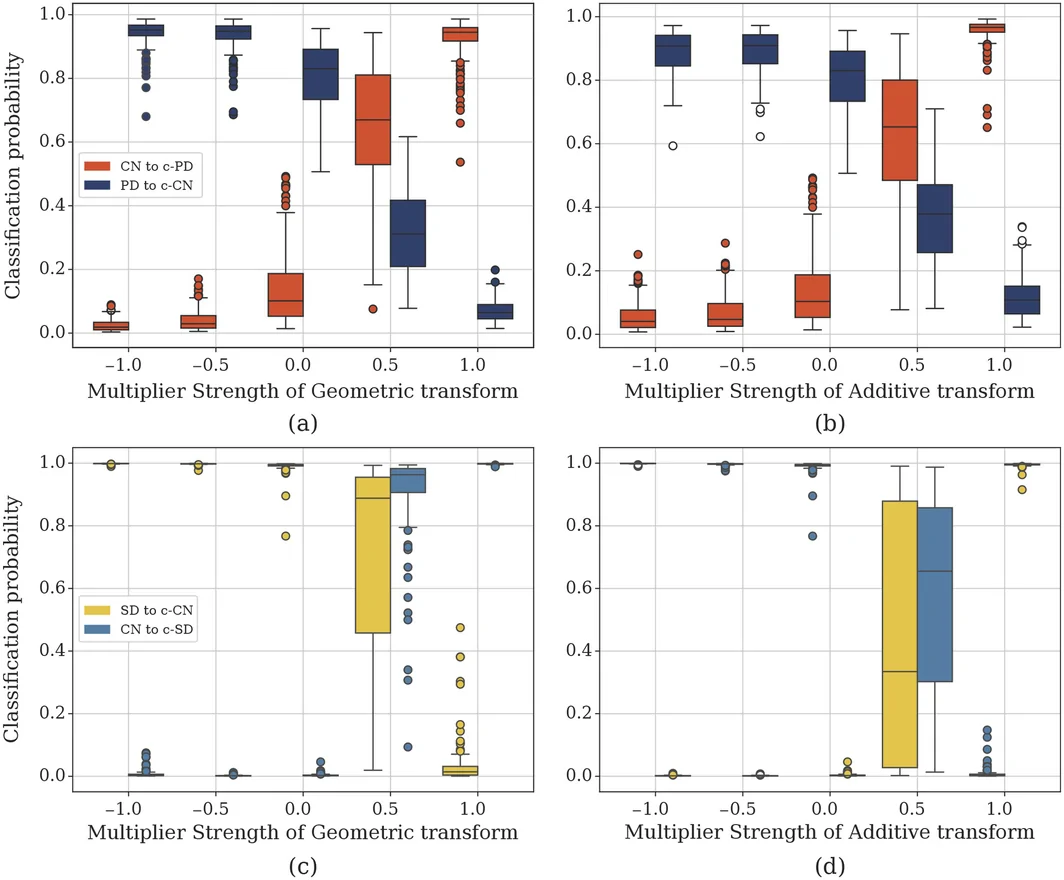
Box‐plots of classification probabilities with varying strengths of the predicted counterfactual transforms for (a) geometry‐constrained and (b) unconstrained counterfactual generation models for $\text{Classifie}r_{\mathrm{PD}}$. Similar plots are also shown for (c) geometry‐constrained and (d) unconstrained models for $\text{Classifie}r_{\mathrm{SD}}$.

The predicted counterfactual transforms demonstrate a stable relationship with the predicted classification probabilities for both $\text{Classifie}r_{\mathrm{PD}}$ and $\text{Classifie}r_{\mathrm{SD}}$, as shown in Figure 7. As the strength of the applied counterfactual transforms increases from 0 to 1, we observe a monotonic‐like change in the classification probabilities in the direction of the respective target classes. This stable transition in classification decision also indicates that the classifiers learnt disease profiles based on severity of respective additive and morphometric features.

Although not a part of the training, the counterfactual models implicitly captured the inverse conversion direction with inverted strength of the counterfactual transforms. As observed in Figure 7, by applying inverted strength (decreasing from 0 to −1) of the counterfactual transforms, the classification probabilities move further towards the original classes. This property of the explained transforms indicates their generalization in representing the spectrum between healthy and diseased states learnt by the classifiers.

### 3.5 Instance‐Level Explanations

**FIGURE 8:**
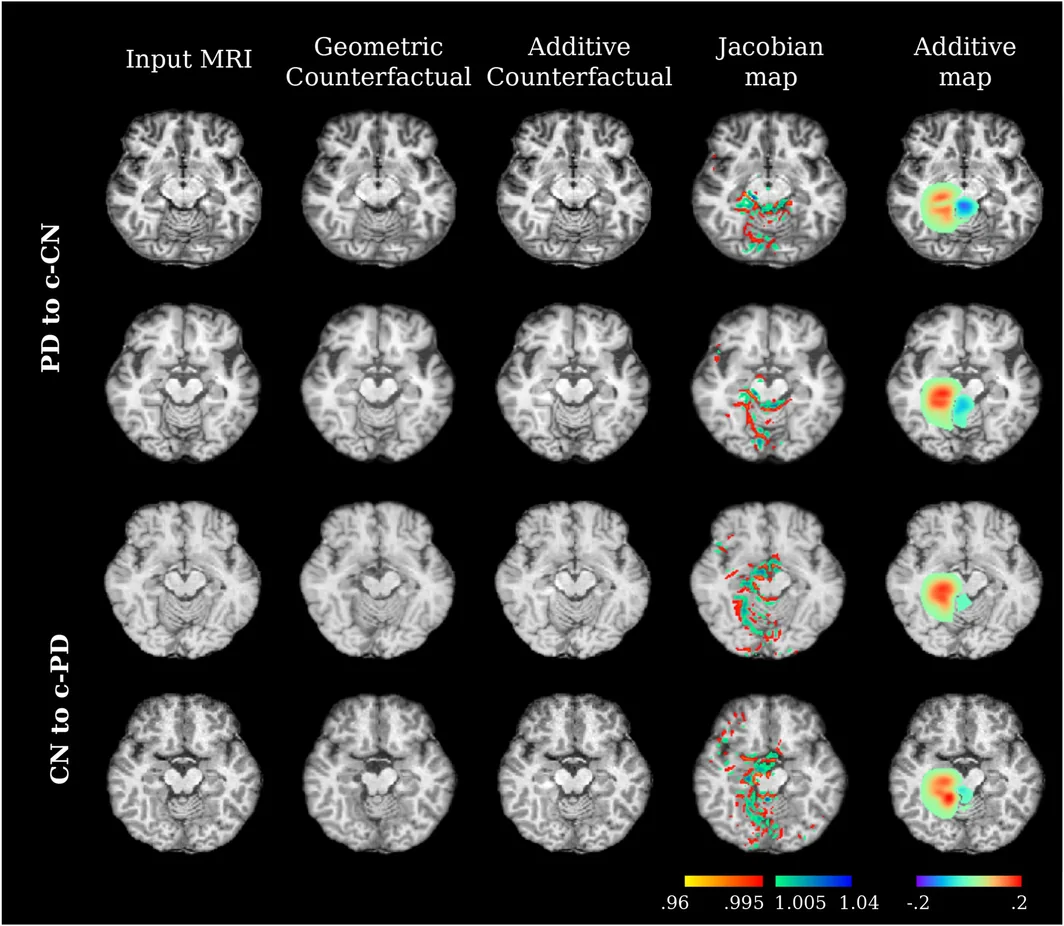
Counterfactual image instances generated using the geometry‐constrained and unconstrained counterfactual generation method for $\text{Classifie}r_{\mathrm{PD}}$.

The geometric changes performed by the trained $\mathrm{GCC}G_{\mathrm{PD}}$ model were generally found to be acting in opposing directions, as shown in Figure 8. For example, an expansion in the brainstem was observed for converting CN image instances to c‐PD, whereas a contraction in the same region was observed for converting PD instances to c‐CN. Observed similarity in the regions targeted by the GCCG and UCG methods shows consistency in modified regions considered important by both methods. Counterfactual image instances for SD counterfactual generation models can be found in Appendix C.

### 3.6 Feature Attribution Explanations

**FIGURE 9:**
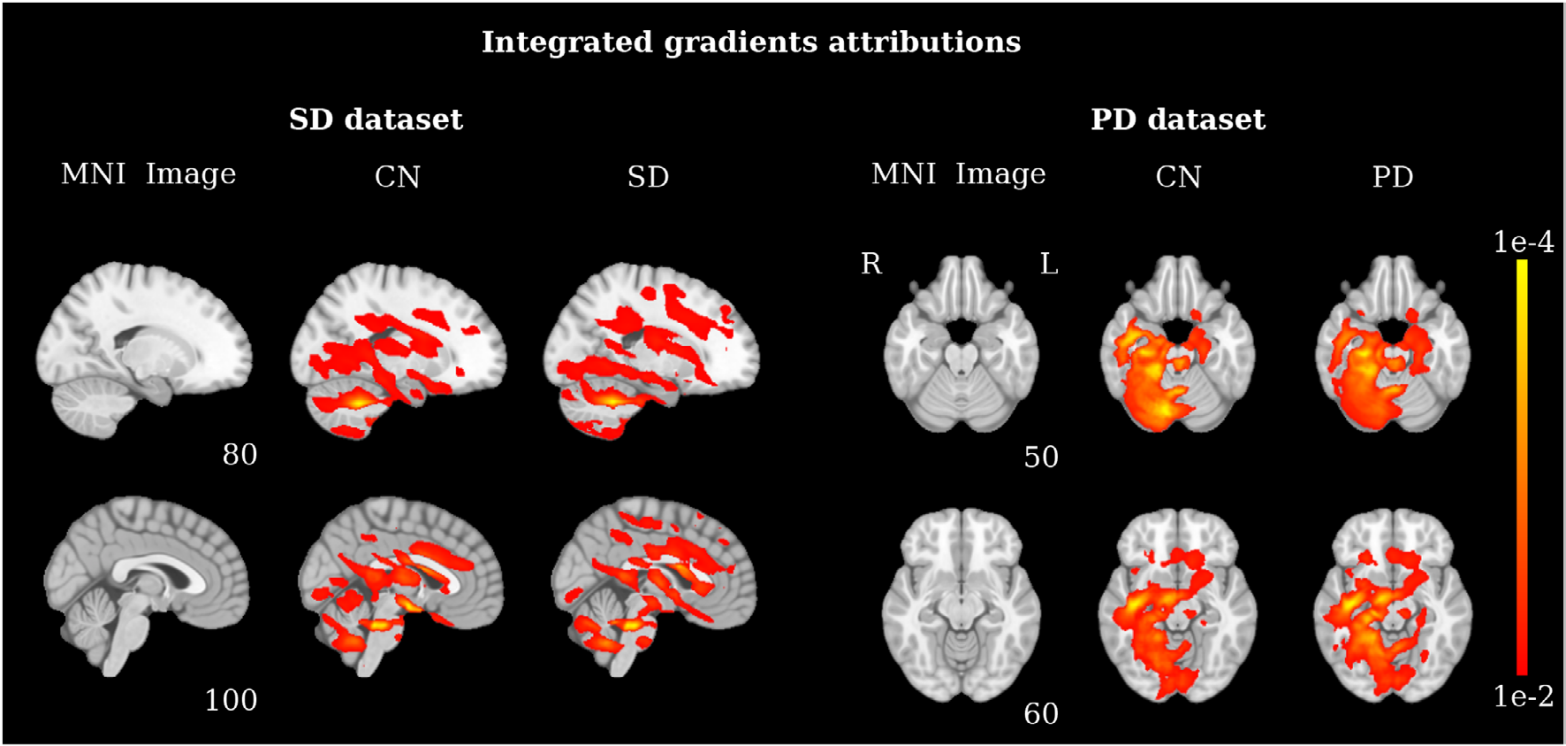
Integrated gradient attributions displayed on the MNI152 template for CN and SD for $\text{Classifie}r_{\mathrm{SD}}$, and CN and PD for $\text{Classifie}r_{\mathrm{PD}}$. Only voxels with positive attributions greater than $10^{-2}$ are shown as colours.

The average explanations produced using Integrated Gradients (Sundararajan et al. 2017), a feature attribution method, registered to MNI152 space for both PD and SD classifiers are shown in Figure 9. The regions with positive attributions above 0.01 are displayed for both classifiers, representing brain regions that were considered important for classification.

### 3.7 Average Transform Properties

The counterfactual image properties of validity, fidelity, and proximity were computed for images generated using the average counterfactual transforms from both generation methods. The average transforms were able to convert all CN images to c‐PD for the testing PD dataset. The geometry‐constrained method had a slightly higher conversion rate of 95.4% than the unconstrained method for converting PD images to c‐CN. Additionally, FID and MS‐SSIM metrics were found to have fidelity comparable to instance transforms with less than 2% change in intensity values as measured by the proximity of the counterfactuals generated by using the average transform. The values for these three metrics are reported in Table 8.

**TABLE 8:** Validity, fidelity, and proximity measured for counterfactual images generated using average transforms for geometry‐constrained ($\mathrm{GCC}G_{\mathrm{PD}}$) and unconstrained ($\mathrm{UC}G_{\mathrm{PD}}$) counterfactual generation methods.

	Conversion rates		Fidelity		Proximity
	CN to c‐PD	PD to c‐CN	FID	MS‐SSIM	
$\mathrm{GCC}G_{\mathrm{PD}}$	100	95.40	0.0009	0.81	1.1e‐2
$\mathrm{UC}G_{\mathrm{PD}}$	100	91.8	0.0011	0.80	9.2e‐3

The classification conversion rates for $\text{Classifie}r_{\mathrm{SD}}$ with the averaged counterfactual transform were found to be lower than the instance transforms. The average maps generated from the unconstrained model achieved higher conversion rates than the geometry‐constrained models, as shown in Table 9.

**TABLE 9:** Classification conversion rates for counterfactual images generated using average transforms for counterfactual generation models on the simulated disease test dataset.

	Conversion rates	
	CN to c‐SD	SD to c‐CN
$\mathrm{GCC}G_{\mathrm{SD}}$	89.2	37.7
$\mathrm{UC}G_{\mathrm{SD}}$	100	83.49

### 3.8 Average Counterfactual Changes

**FIGURE 10:**
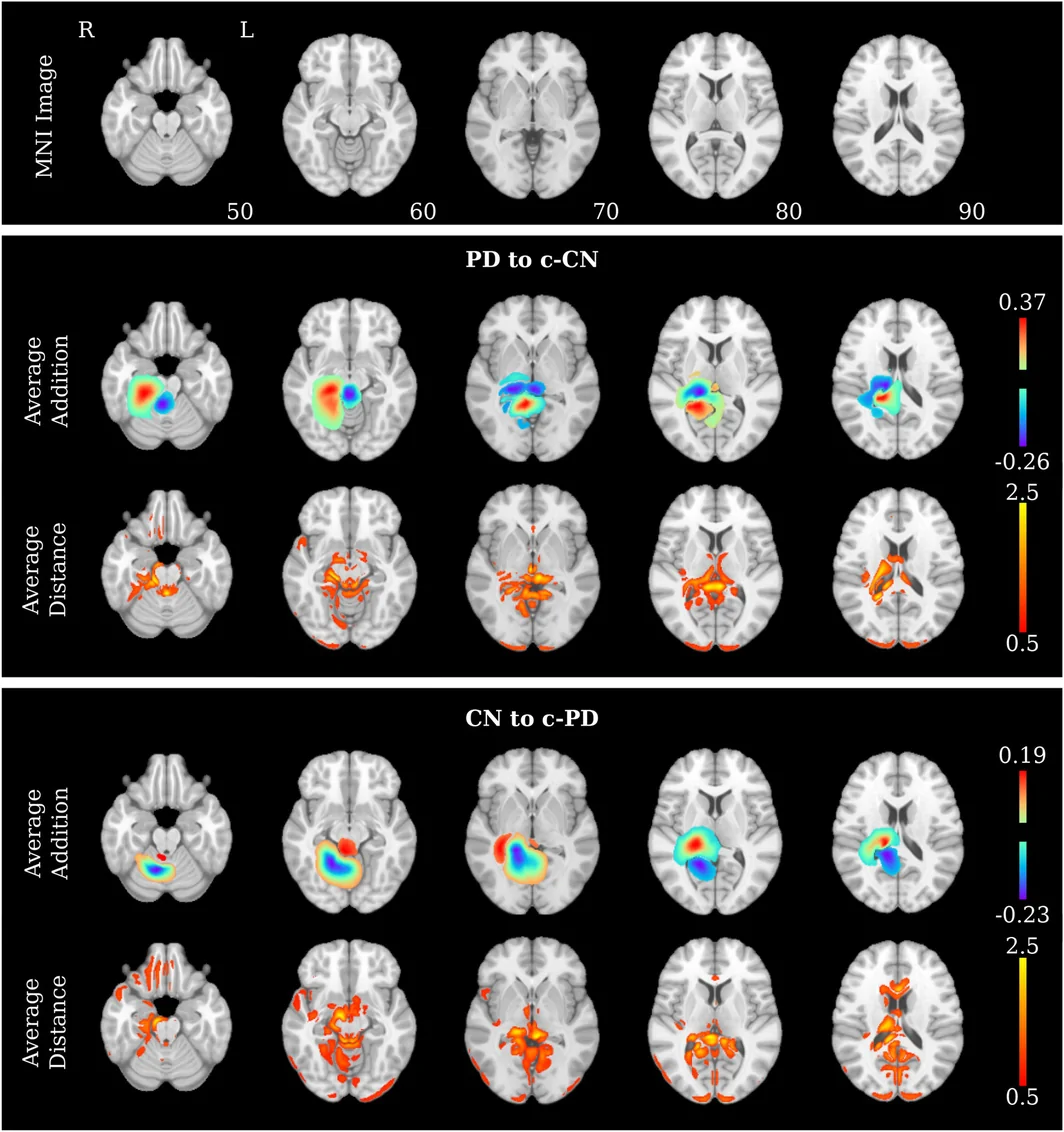
Average counterfactual displacement magnitude and additive transforms displayed over MNI152 template for $\mathrm{GCC}G_{\mathrm{PD}}$ and $\mathrm{UC}G_{\mathrm{PD}}$ respectively. The average transforms predicted in PD train dataset were computed for PD images converted to classified control (c‐CN) and CN images converted to c‐PD as classified by $\text{Classifie}r_{\mathrm{PD}}$.

The average of the transforms predicted by the $\mathrm{GCC}G_{\mathrm{PD}}$ and $\mathrm{UC}G_{\mathrm{PD}}$ counterfactual models performed changes in mainly the same regions as seen in average additive and displacement maps in Figure 10. The scalar additive changes were averaged for every voxel location after applying the spatial transformations obtained from registration. This is shown, together with the magnitude of the average of registered displacement vectors, in Figure 10. The average geometric and additive maps highlighted that the changes in the brainstem, cerebellum, and ventricles contributed towards the classification decision.

**FIGURE 11:**
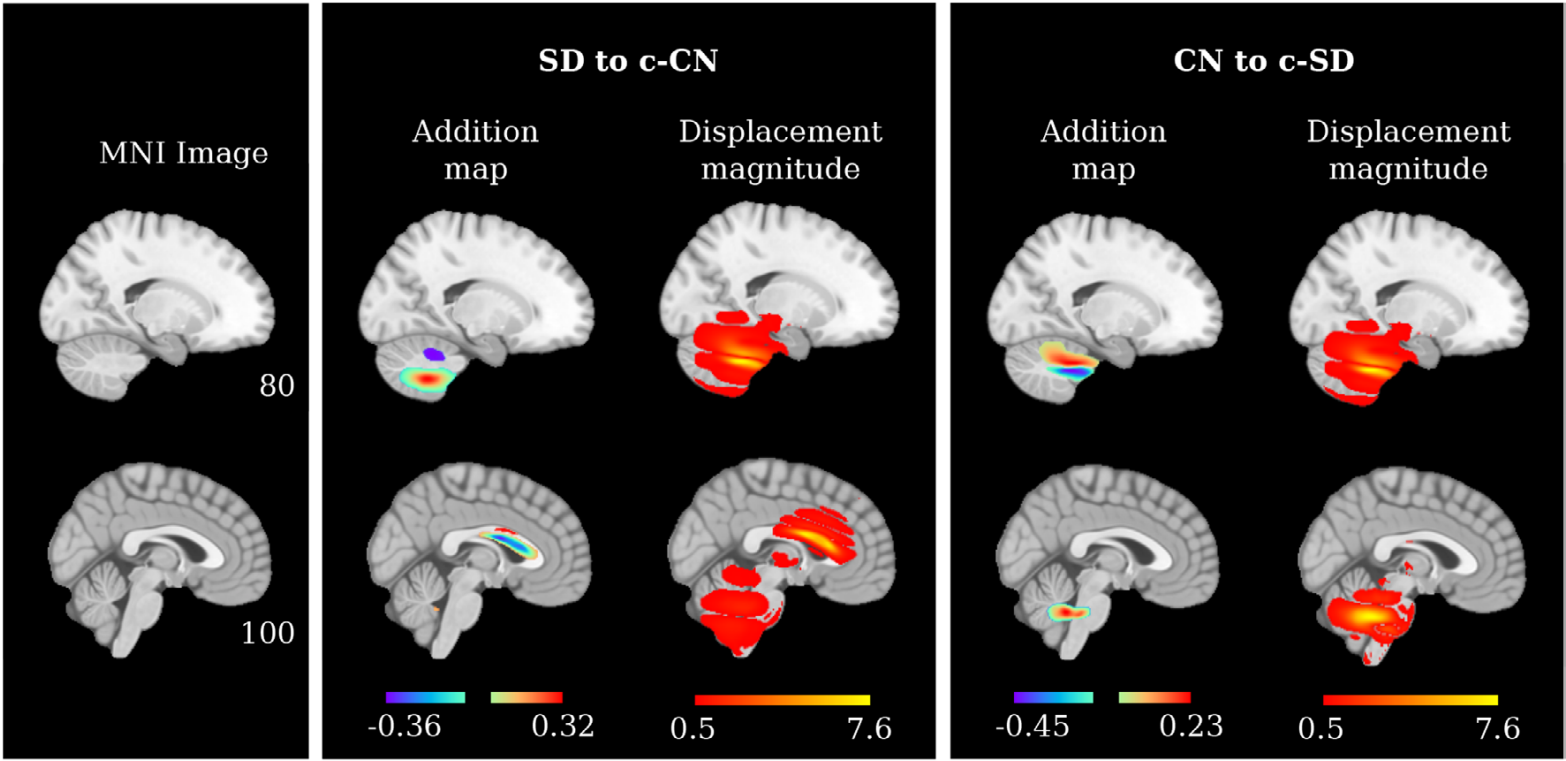
Average of additive and displacement counterfactual transforms predicted by $\mathrm{UC}G_{\mathrm{SD}}$ and $\mathrm{GCC}G_{\mathrm{SD}}$ models, displayed over the MNI152 template.

For the SD models, the average maps shown in Figure 11 highlight changes performed at regions that are consistent with the introduced disease features. To convert images from CN to c‐SD, only one of the two disease features was explained by the counterfactual transforms, whereas both the disease features were represented in SD to c‐CN counterfactual conversion. Moreover, the changes represented by the average displacement maps can be qualitatively observed to be more representative of the simulated features (as described in Section 2.2) in comparison to the additive changes.

#### 3.8.1 Average Volumetric Changes

**FIGURE 12:**
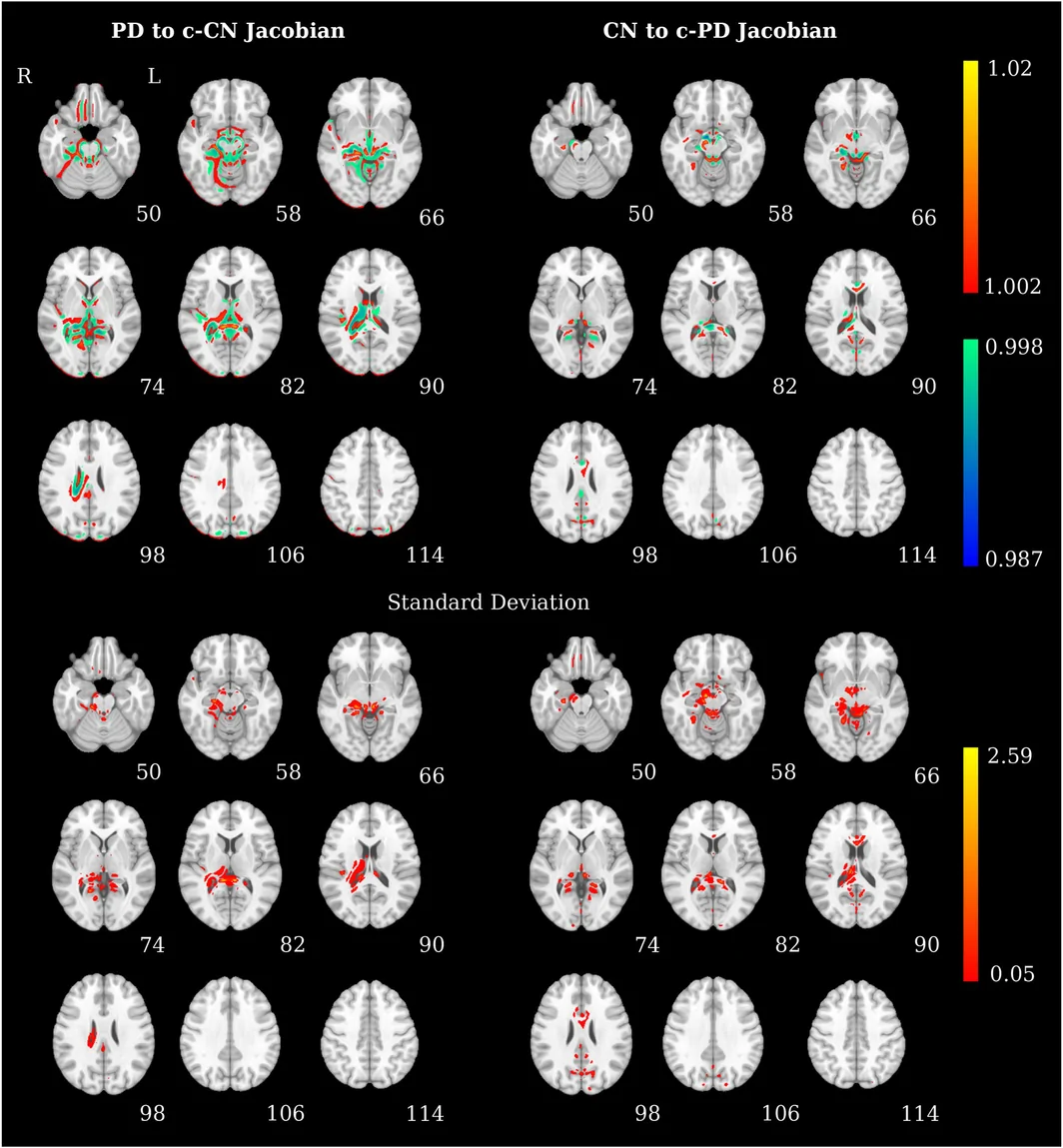
Jacobian maps and their voxel‐wise standard deviation computed for the average displacement counterfactual transforms predicted by the $\mathrm{GCC}G_{\mathrm{PD}}$ model showing brain regions that were expanded or contracted to convert the classification decision of $\text{Classifie}r_{\mathrm{PD}}$.

The average volumetric change performed by the $\mathrm{GCC}G_{\mathrm{PD}}$ model for anatomical locations in MNI152 space, computed using the Jacobian of the average displacement maps, is shown in Figure 12. The brainstem and ventricles were observed to be contracted for average PD to c‐CN transform, whereas they were expanded on average for CN to c‐PD conversions. This opposing volumetric change can also be observed for other regions transformed by the predicted deformation maps. Additionally, the voxel‐wise standard deviation of the Jacobian maps highlights regions exhibiting high average volumetric changes also display relatively higher standard deviations. However, low standard deviations are observed for most anatomical regions.

## 4 Discussion

Explainability is an important consideration for clinical and research applications of “black‐box” deep‐learning classifiers. Many types of explanation for classification decisions fail to provide an understanding of how disease‐specific information is used in the classification. In our work, we provide an explanation that is specific to morphological changes in input images that contribute towards decisions made by a classifier, as pathological shape changes in anatomy are a hallmark of multiple diseases, including complex neurodegenerative disorders. In addition to the explanations provided for image instances, we also provided an explanation of group‐level morphological features that were used by the classifier, on average, to predict image labels. For a PD classifier, the computed average explanations highlighted common morphological brain changes, such as atrophy in the brainstem, that were being used by the classifier at a group level, in a standard anatomical brain space. This can provide domain experts with an interpretable geometric explanation of the disease profile learned by the classifier.

Explanations provided by Integrated Gradients had limited interpretability due to unclear anatomical boundaries and lack of clarity in the types of changes being considered important for classification. For the simulated disease classifier, the average feature attribution maps (shown in Figure 9) highlighted regions where morphological changes had been introduced as well as regions where no changes were introduced. These were both, according to this method, important to the classifier, which is potentially due to spurious patterns and correlations learnt from the training datasets. It is also possible that it highlights areas of healthy brain anatomy that are important for the classifier to obtain a reference frame for the anatomy as a whole. This demonstrates that the interpretation of why a region was considered important could not be inferred directly from the feature attribution maps. Whereas the average displacement maps generated from the proposed method, shown in Figure 11, were able to highlight regions that were considered important due to their morphology. Furthermore, the average displacement maps were found to be more aligned with anatomical boundaries, as opposed to the dispersed feature attribution maps that spanned larger volumes, spread across multiple brain regions. The geometric explanations provided by the proposed method showed improved anatomical localization in explanations produced for classifiers trained on SD and PD datasets. The proposed method, applied to PD and SD datasets, showed an improvement in anatomical localization of explanations. Further testing on other datasets can be performed to establish whether this localization is a general feature of this approach.

To ensure meaningfulness of the derived explanations, the properties of validity, fidelity, and proximity were verified for the produced counterfactual images. These properties ensured that the images were generated by performing minimal and realistic modifications that effectively changed the classification decision, which are important properties to support the biological plausibility of the transforms. Furthermore, the counterfactual transforms were shown to utilize stable features that allowed for smooth changes in classification predictions with varying strengths of the transforms, as observed in Figure 7. It was also established that the morphological changes preserved topology by verifying that the Jacobian determinants were greater than zero for all predicted displacement maps, which is important to ensure plausibility of spatial deformations. These properties support the claim that meaningful counterfactual transforms were generated to perform modifications that are representative of disease‐related differences present in the training datasets.

It is also necessary to try to establish if the proposed explainability method is correctly reporting what the classifier was using internally. Results from various experiments reported above corroborate this. In particular, the morphological features for the simulated disease, as explained by the method for $\text{Classifie}r_{\mathrm{SD}}$ and shown in Figure 11, were found to be similar to the ground truth features, shown in Figure 1. Although it is possible that the classifier was not using these features, the fact that the classifier accuracy and conversion rates were both extremely high strongly supports the assertion that the explainability method is showing the real information that is used internally in the classifier. In addition, atrophy in the brainstem is a known hallmark of PD, which was also explained by the proposed method as a morphological feature contributing towards the decisions made by $\text{Classifie}r_{\mathrm{PD}}$. The necessity and sufficiency experiments, presented in Table 7, revealed that while deformations in no single brain region were necessary to change the $\text{Classifie}r_{\mathrm{PD}}$ decisions, the template region containing the brainstem demonstrated the highest sufficiency for changing the classification. Furthermore, these regions were included within the outputs of independent explainability methods: Integrated Gradients and the UCG method. Given all of the above evidence it is likely that the classifiers were using these disease‐related features, and this indirectly validates that the proposed method is correctly interrogating the classifier.

The geometric explanations were found to be more interpretable than the explanations produced by the unconstrained method since the former are limited to changes in morphology. Despite the ability of unconstrained counterfactual generation to perform both geometric and non‐geometric changes, similar brain regions were modified by the UCG and GCCG methods. Furthermore, the explanations produced by the geometry‐constrained method were qualitatively found to be more localized with respect to the anatomy in contrast to the diffused additive changes from the unconstrained method, as observed in Figure 8. These initial results indicate that a constrained generation method, such as the proposed GCCG method, provides both a more interpretable and more precise understanding of anatomical aspects important to the classifier compared to a more flexible, unconstrained generation method.

Instance geometric explanations, although informative, cannot be directly used to explain the general disease features learnt by a classifier. Without a general explanation of the learnt disease, a user would have to undertake a non‐trivial task of observing hundreds of instance explanations (such as the one shown in Figure 8) and mentally tracking similarities across all the 3D images to find common patterns. By observing the average geometric counterfactual changes registered to MNI152 space, as shown in Figures 10 and 11, a clear interpretation of the learnt morphological features can be understood immediately. The average displacement maps shown for $\text{Classifie}r_{\mathrm{PD}}$ explain that atrophy of the brainstem and surrounding regions are general disease features learnt by the classifier.

The average maps can also reveal heterogeneity in learnt disease features, which is prevalent in most neurological disorders. This heterogeneity can arise due to a varied combination of strength, extent, and location of the pathological brain changes in different individuals. If the same change was present in each individual then the average maps should have the same conversion rate as the instance maps. However, when there is a substantial reduction in the conversion rates between instances and the average, it indicates that either some heterogeneous features in instance maps were lost in the averaging process or that multiple such features were retained in the maps but are not normally present together in individual instances. The presence of heterogeneity is particularly emphasized in our results by the > 60% difference between average and instance conversion rates for $\text{Classifie}r_{\mathrm{SD}}$, where the simulated disease features were designed to be heterogeneous. This difference was more prominent for $\mathrm{GCC}G_{\mathrm{SD}}$ than for $\mathrm{UC}G_{\mathrm{SD}}$. This variance could be attributed either to noisy additive counterfactuals resulting from a lack of mathematical constraints, or to spurious, non‐morphometric changes learned by $\text{Classifie}r_{\mathrm{SD}}$ alongside the true differences. Furthermore, a minimal reduction in conversion rates was observed for healthy to disease conversion (as opposed to the disease to healthy conversion rates) for both PD and SD counterfactual models. Since only one of the multiple changes is required to make an image appear diseased, whereas an individual‐specific subset of changes is required to remove relevant disease aspects, disease to healthy conversion for instance transforms were more varied but captured the specifics of the disease changes better. However, gaining an overall picture of the disease changes is still easier to view in the average transforms.

### 4.1 Limitations

Although the proposed geometry‐constrained counterfactual generation has its advantages, it is not without limitations. The geometric explanations do not capture micro‐structural brain changes that are usually reflected in intensity changes, making the proposed explainability method unsuitable for diseases with predominantly non‐morphological anatomical changes. Further research is required to adequately explain these constrained intensity related changes along with the spatial deformations using counterfactual methods.

Additionally, while the proposed counterfactual generation method provided an instance explanation of a classification using a specific image transform, it is not the only possible explanation that could convert the classification decision. Multiple valid counterfactual changes can possibly be generated for converting the classification decision that may or may not have common features depending on the disease heterogeneity and the trained classification model.

Finally, a fundamental limitation applicable to our framework, as well as most post hoc explainability methods, is the difficulty in establishing an internal verification of how the classifier utilizes the generated counterfactual features. Due to the inherent complexity and highly non‐linear nature of deep learning architectures, we could not directly trace the network's internal mathematical routing to a specific anatomical change. Therefore, while our validation experiments demonstrate that the identified image features are robustly associated with classification decisions, they are not a verified proof of the network's internal logic.

### 4.2 Future Directions

Although our approach was validated on 3D T1‐weighted MRIs, the geometric counterfactual framework can be naturally extended to classifiers trained on other structural imaging modalities, not limited to MRIs.

Another possible methodological extension is isolating and exploring specific intensity‐related tissue changes after the geometric transformations have been applied. Decoupling the counterfactual explanations into separate geometric and intensity components could provide granular, more interpretable explanations to domain experts.

Additionally, characterizing heterogeneity in neurodegenerative disorders like Parkinson's Disease remains a challenge. By generating a diverse set of counterfactual variations for a single input image, future work could explore clustering of the resulting deformation fields. This approach could uncover distinct, prototypical disease subtypes learnt by the classifier, offering valuable insights into population‐level heterogeneity.

Finally, the idea of generating constrained counterfactuals could be adapted to diverse non‐geometric constraints to explain relevant information for different classifiers (such as maintaining functional connectivity). Furthermore, composing multiple such independent, constrained counterfactuals could be utilized to provide a disentangled understanding of different facets of complex classification decisions.

## 5 Conclusion

We presented a geometrically constrained counterfactual explainability method that reveals morphological features learnt by structural MRI classifiers. By restricting the explanations to geometric transformations, the method provides anatomically relevant insights into how morphological information was used by the classifiers. By showing that the explanations generated for a highly accurate simulated disease classifier were closely aligned to the synthetic ground truth, we indirectly demonstrate the efficacy of the method in correctly explaining the classifiers. Using PD as an example, we demonstrated that the method identifies clinically relevant atrophy patterns and enables both instance‐ and group‐level interpretation of learnt disease features. This work provides a foundation for improved interpretability of morphological contributions in deep‐learning classification, which is important in building trust for clinical adoption of deep‐learning models.

## Funding

Funding for this study was provided as research scholarship from Adelaide University.

## Figures and Tables

**FIGURE A1:**
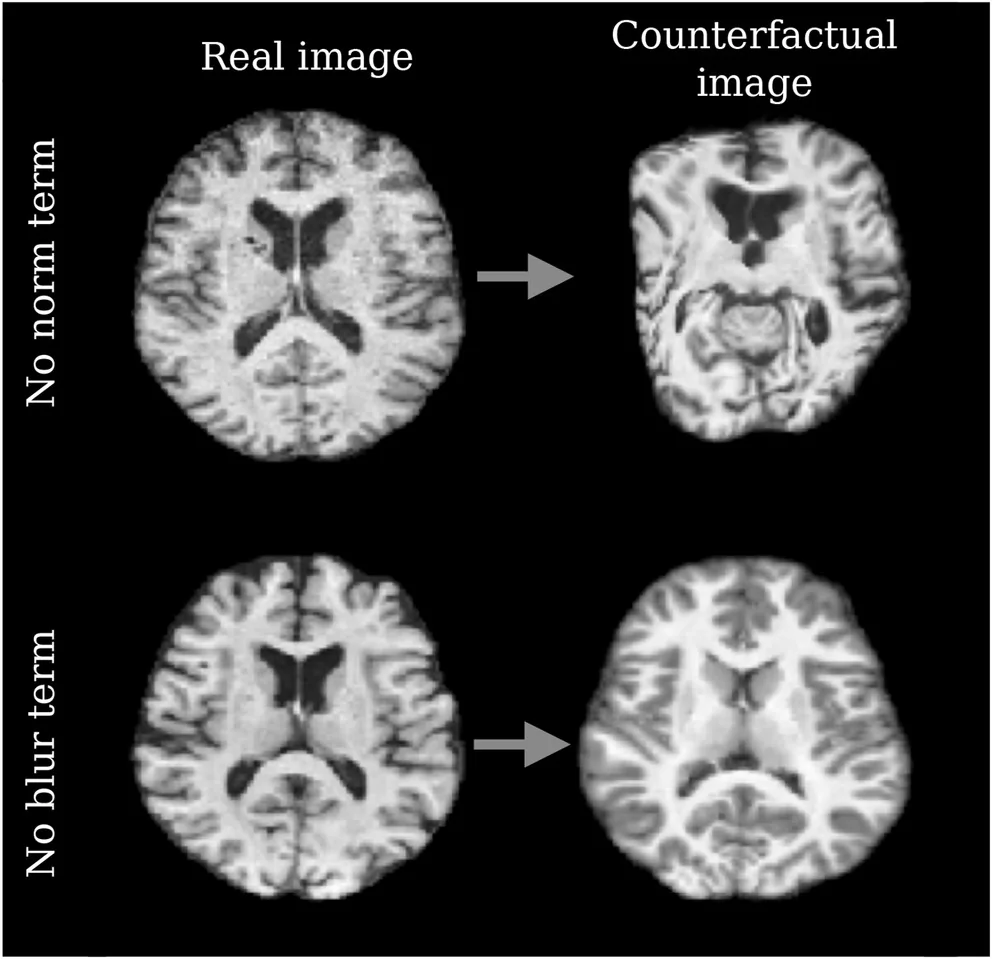
Counterfactual images generated by models trained for ablation study.

**FIGURE A2:**
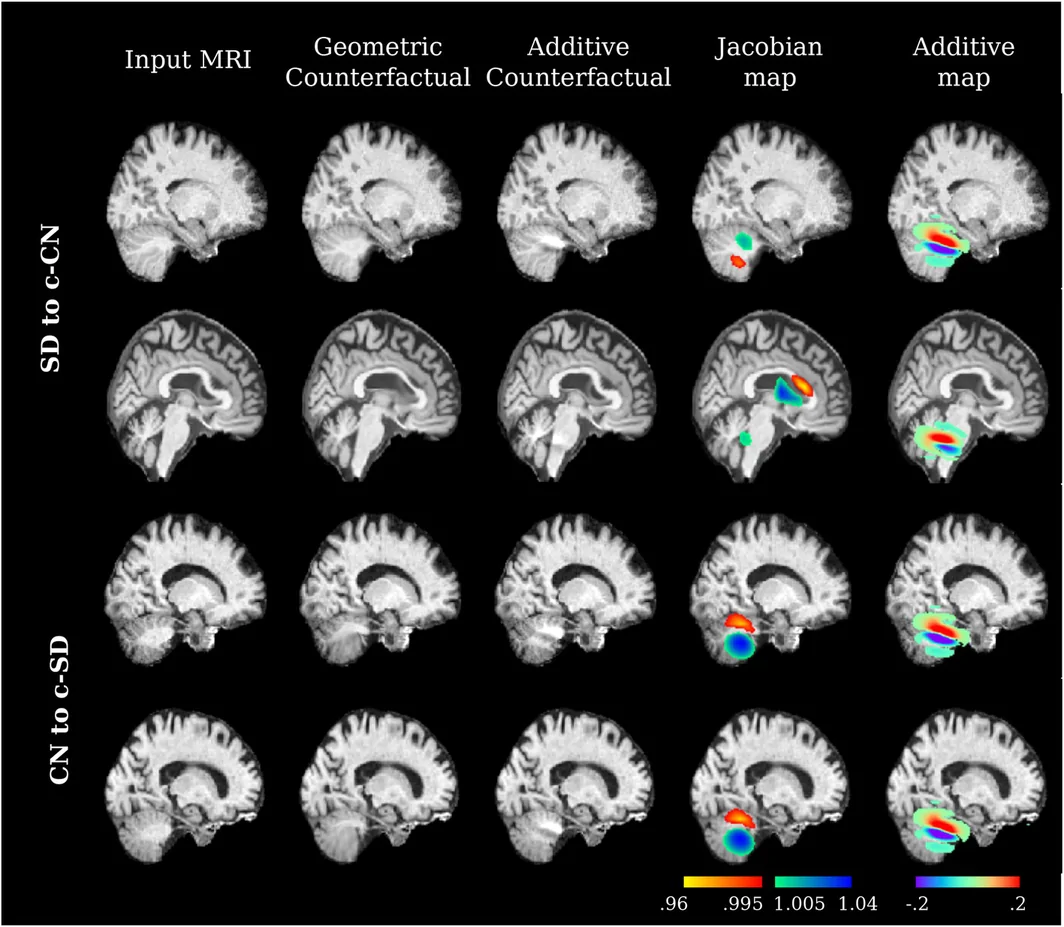
Counterfactual image instances generated using the geometry‐constrained and unconstrained counterfactual generation method for $\text{Classifie}r_{\mathrm{SD}}$.

**FIGURE A3:**
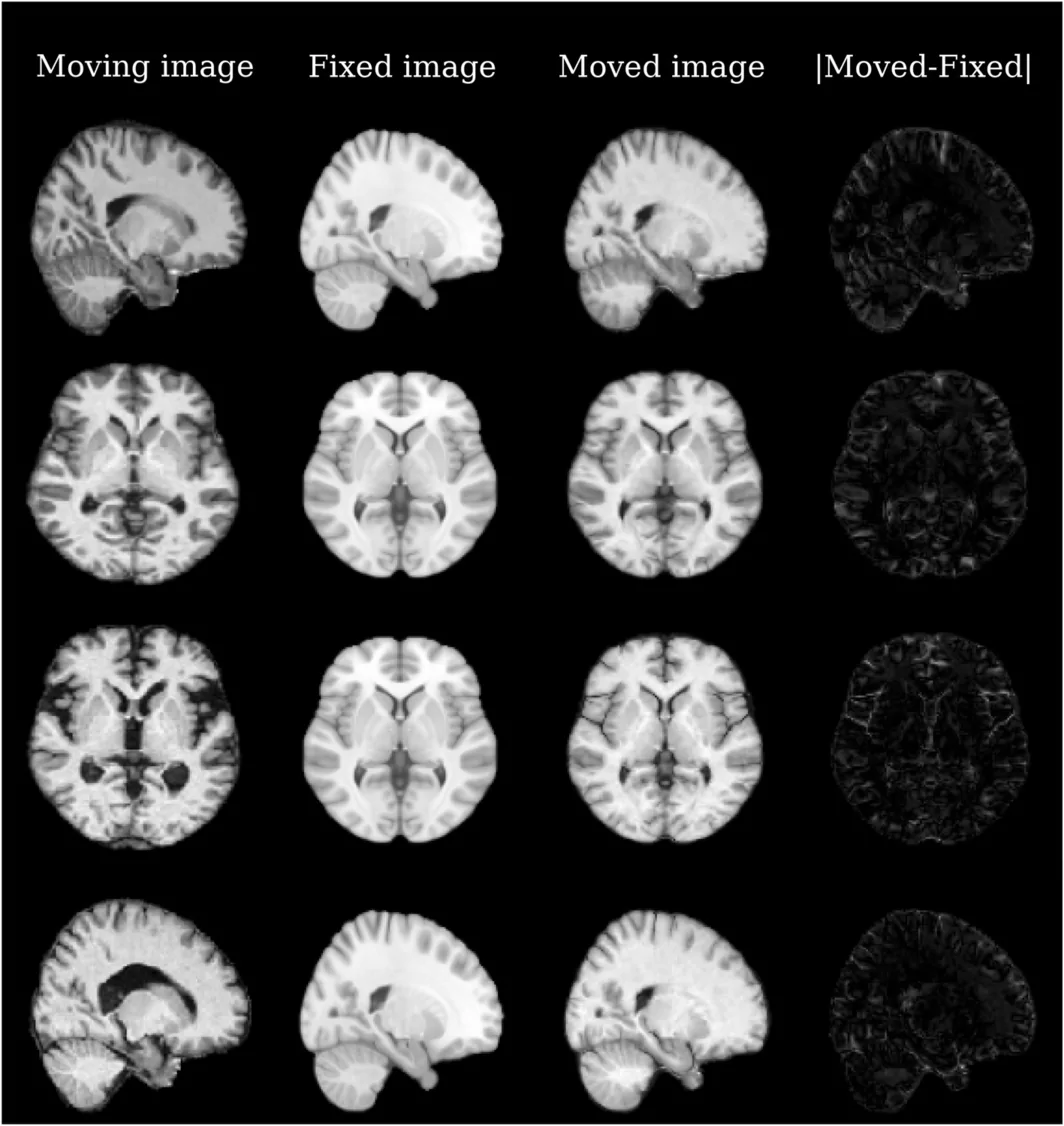
Registered image examples using the trained registration model where moving images were registered to the MNI152 template.

**FIGURE A4:**
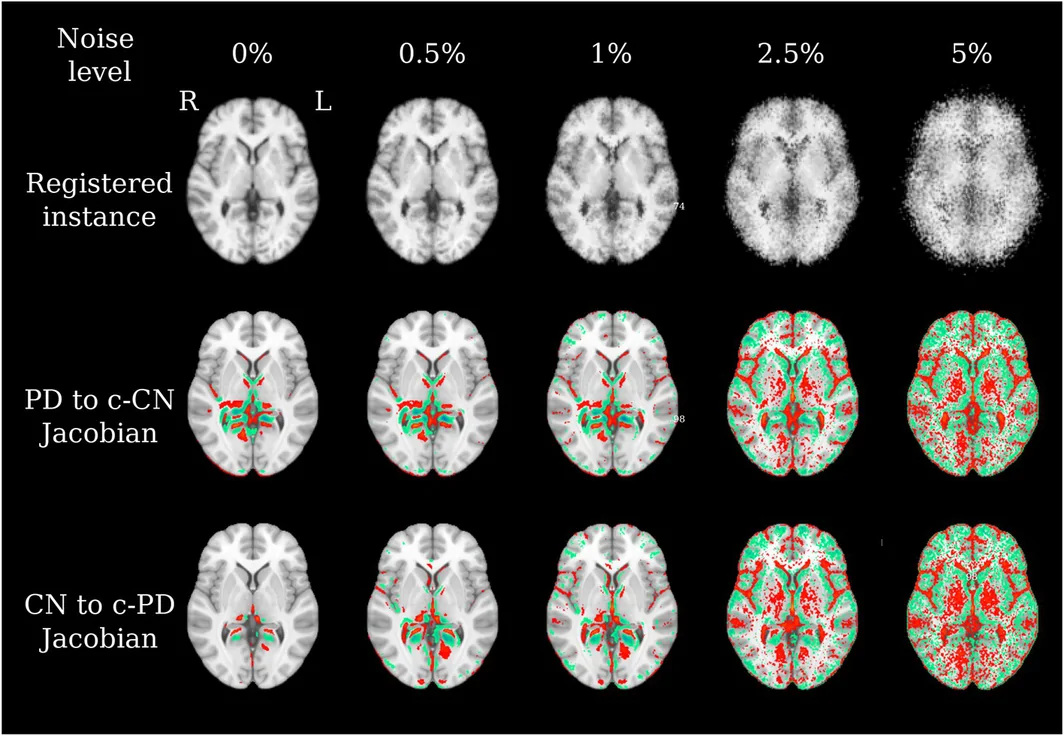
Average deformation maps obtained for $\text{Classifie}r_{\mathrm{PD}}$ by introducing varying levels of noise in the instance registration fields.

## Data Availability

The data that support the findings of this study are available from following studies: PPMI, ADNI, and OASIS. Restrictions apply to the availability of these data, which were used under license for this study. The data is available upon request from individual studies.

## Appendix A Simulated Disease Features

The simulated disease features were applied by generating radial Gaussian displacement fields which could either expand or shrink the contents from center of a chosen image sub‐volume, similar to blowing or shrinking a balloon. To generate the expanding displacement field for the ventricle (SD feature 1), we generated a 40×40×40 displacement field using normal continuous random variable N as implemented in Scipy library with mean μ=0 and variance σ=6. The magnitude of displacement vector for every voxel in the sub‐volume was computed as $6\times N\left(d_{c}|\mu =0,\sigma =6\right)$ for x,y, and z directions, where $d_{c}$ is euclidean distance of the voxel from centre of the sub‐volume, where N was computed using Equation (A1). Since the spatial transformer implementation scales the image dimensions in $\left[-1,1\right]$ range, and the computed displacement magnitudes operated in sub‐volumes of side length of 40 voxels, the voxel displacement magnitudes can be computed by multiplying 40/2 to the scaled output displacements.

Since the magnitude of the vector depended only on its distance from the center, the displacement vectors were generated facing radially outwards from the center of the sub‐volume. To smoothen the artefacts on the edges of the displaced sub‐volume, we then linearly scaled the magnitude of displacement vectors on the edges to reach 0 for the final 10% of the sub‐volume boundary. Finally, this pull displacement field was applied to affine registered, pre‐processed images at voxel location (65, 80, 60) as center of the displacement field to generate SD feature 1 images, as shown in Figure 1.

(A1)
$$N\left(d_{c}|\mu =0,\sigma =6\right)=\frac{\exp\left(-\frac{1}{2}{\left(\frac{d_{c}-\mu }{\sigma }\right)}^{2}\right)}{\sqrt{2\pi }\sigma }.$$

Similarly, the SD feature 2 images were generated using 40×40×40 displacements generated using same Gaussian parameters with their direction reversed by multiplying the vectors with −1. The generated pull displacement field was also smoothened with 10% margin as described earlier for SD feature 1. This displacement field centered at voxel location (50, 45, 25) was applied to pre‐processed images to generate the SD feature 2 images for the simulated dataset.

## Appendix B Generator Ablations

The ablation results for loss terms used in generator training indicate improved fidelity and stable training when all terms were present in training, as shown in Table A1. Additionally, counterfactual image examples generated for the ablation models are shown in Figure A1.

**TABLE A1:** Ablation results for introduced multiplicative (Mult), norm regularization ($L_{\mathrm{norm}}$), and global structure preservation ($L_{\text{blur}}$) terms in the generator loss.

Model	Mult	$L_{\mathrm{norm}}$	$L_{\text{blur}}$	FID	Proximity
$\mathrm{GCC}G_{\mathrm{PD}}$	No	Yes	Yes	NA^a^	NA^a^
	No	No	Yes	0.099	0.096
	No	Yes	No	0.0015	0.056
	Yes	Yes	Yes	0.0007	9.4e‐3

^a^ Unstable training that did not converge.

## Appendix C Simulation Counterfactual Instances

The counterfactual images generated by geometry‐constrained and unconstrained counterfactual generation methods trained for $\text{Classifie}r_{\mathrm{SD}}$ show structural changes being performed by both geometric and intensity changes to generate the images. The image instances presented in Figure A2 show counterfactual changes being performed at the locations of simulated disease features. Moreover, although structural changes can be performed by intensity transforms, the geometric transforms observed to qualitatively represent the structural changes better than additive transforms as observed for the simulated features near the ventricles.

## Appendix D Registration Model

### Image Registration Model

We trained a deformable image registration model to register images to a target image space and obtain corresponding registration displacement fields. The registration model was trained using the hierarchical transform architecture, similar to the generator, which was adapted from methods presented in Balakrishnan et al. (2019) and Strittmatter and Zöllner (2024). The network was trained by optimizing mean squared error between moving ($x_{\text{move}}$) and target ($x_{\text{target}}$) images and the smoothing regularization term similar to $L_{\mathrm{norm}}$ as used in the geometry‐constrained counterfactual generator. The hyper‐parameter $\alpha_{r}=100$ was used for the smoothing regularization term.

(D2)
$$L_{\mathrm{Reg}}={\left\|x_{\text{move}}-x_{\text{target}}\right\|}^{2}+\alpha_{r}\sum_{i,j,k}{\left\|\nabla \phi_{i,j,k}\right\|}^{2}.$$

Despite availability of multiple registration models based on non‐deep learning and deep learning methods, we trained our own registration model to obtain displacement fields that are in a similar format to the deformations used by the counterfactual transforms. This consistent representation of deformation fields across models allows for combined use of fields for group‐level explanations as explained in the following section. The trained model achieved a mean squared error of 0.0018 between moving and fixed images in a test set. Some examples of the images trained using the registration model are shown in Figure A3.

### Vector Field Transformation

To create an average displacement map it is necessary to transform a vector field from one space to another, and this differs significantly from how a scalar image is transformed. The reason for this is that it is not sufficient to simply shift the location of the vector but that the vector's magnitude and direction also need to be modified. This is most easily understood and implemented by considering that the vector's start and end points are transformed by different parts of a non‐linear transformation, so that their relative distance apart and orientation are going to change. More specifically, if there is a vector $v\left(x\right)$ subject to a spatial transformation ϕ then the new vector will be located at $x^{\prime }=\phi \left(x\right)$ and be defined as $v^{\prime }\left(x^{\prime }\right)=\phi \left(v\left(x\right)+x\right)-\phi \left(x\right)$, being the difference between the start and end points.

In the case of creating a version of the counterfactual transformation in an average (MNI) space, we start with two transforms: $\phi_{C}$, the counterfactual transformation $A\to A^{c}$; and $\phi_{M}$, the transformation to MNI space A→M. The goal is to create a transformation from the MNI‐space version of the image that recreates the counterfactual version of the image in MNI‐space. Given that coordinate $x_{a}$, in image A, is mapped to its corresponding MNI coordinate version, $x_{m}$, using $x_{m}=\phi_{M}\left(x_{a}\right)$, and to its counterfactual version, $x_{c}$, with $x_{c}=\phi_{C}\left(x_{a}\right)$, then applying the MNI transformation to $A^{c}$ would result in $x_{c}\to x_{\mathrm{mc}}$ where $x_{\mathrm{mc}}=\phi_{M}\left(x_{c}\right)$. Therefore the desired transform maps $x_{m}\to x_{\mathrm{mc}}$, and can be defined as $x_{\mathrm{mc}}=\phi_{\mathrm{MC}}\left(x_{m}\right)=\phi_{M}\left(x_{c}\right)=\phi_{M}\left(\phi_{C}\left(x_{a}\right)\right)=\phi_{M}\left(\phi_{C}\left(\phi_{M}^{-1}\left(x_{m}\right)\right)\right)$. From this it is easy to see that $\phi_{\mathrm{MC}}=\phi_{M}\circ \phi_{C}\circ \phi_{M}^{-1}$, where ∘ denotes function composition.

When working with discrete images and transformations, there is an additional complication for calculating the required function composition. This is that the discrete form for a non‐linear transform is usually represented by precise mappings at grid points and then interpolated between these. Furthermore, to make resampling of images more convenient it is common for the discrete transformation to map from the grid points in the destination space so that the desired intensities at these grid points can be easily determined from interpolation of intensities in the source space. That is, if an image moves from space A→B then the transformation is usually defined by $\phi^{-1}:B\to A$ such that $\phi^{-1}\left(x_{b}\right)=x_{a}$ is easily calculated for every grid point in space B. This is sometimes referred to as a pull representation, as opposed to a push representation that defines the mapping for each grid point in the source space A. It is still straightforward to define how a pull representation maps from any point in space B to the corresponding point in space A, by interpolating the appropriate deformation vectors at the grid points in space B, but the opposite is not true and hence it is necessary to calculate the inverse transformation if the mapping is needed to go from an arbitrary starting point in the source space, A, to the corresponding point in space B.

The representation that is used in our network is a pull representation and, consequently, to get the function composition we require it is necessary to invert the transformations $\phi_{M}$ and $\phi_{C}$ so that we can map easily from any point in A to $A^{c}$ and similarly for mapping points in A to M (whereas M to A is easy with the pull representation we already have). Therefore we calculate the inversions of these spatial transformations, using an algorithm defined in the next subsection, so that we can straightforwardly perform the chain of mappings: $x_{m}\to x_{a}\to x_{c}\to x_{\mathrm{mc}}$.

### Inversion of a Spatial Transformation

To invert a spatial transform we propose an iterative algorithm that finds an approximate inversion by refining local approximations. The algorithm, as shown in Algorithm A1, tries to minimize a cost function computed by applying the approximate inverse to the original transform which should ideally cancel the movements and return identity transform I. We start by approximating the inversion to be an identity transform and for every location we randomly perturb it in two opposing directions, choosing the displacement the minimizes its local deviation from the identity mapping.

ALGORITHM A1 Inverting a pull spatial transform. Given $\phi_{A\to A^{c}}\;\forall \;g_{A}\in A$
Approximate $\phi_{A^{c}\to A}={\phi^{-1}}_{A\to A^{c}}=I$

**for** $g_{A^{c}}\in A^{c}$: $g_{A^{c}}$ is a grid point in $A^{c}$ **do**
 **while** ${\left\|\phi_{A\to A^{c}}\left(\phi_{A^{c}\to A}\left(g_{A^{c}}\right)\right)-g_{A^{c}}\right\|}_{2}>0.005$ **do**
  $\delta \leftarrow {\left\|\phi_{A\to A^{c}}\left(\phi_{A^{c}\to A}\left(g_{A^{c}}\right)\right)-g_{A^{c}}\right\|}_{2}$
  ψ← random unit vector  ${\phi^{\left(+\right)}}_{A^{c}\to A}\left(g_{A^{c}}\right)\leftarrow \phi_{A^{c}\to A}\left(g_{A^{c}}\right)+\psi \frac{\delta }{10}$
  ${\phi^{\left(-\right)}}_{A^{c}\to A}\left(g_{A^{c}}\right)\leftarrow \phi_{A^{c}\to A}\left(g_{A^{c}}\right)-\psi \frac{\delta }{10}$
  **if** ${\left\|\phi_{A\to A^{c}}\left({\phi^{\left(+\right)}}_{A^{c}\to A}\left(g_{A^{c}}\right)\right)-g_{A^{c}}\right\|}_{2}<\delta$ **then**
   $\phi_{A^{c}\to A}\left(g_{A^{c}}\right)\leftarrow {\phi^{\left(+\right)}}_{A^{c}\to A}\left(g_{A^{c}}\right)$
  **else if** ${\left\|\phi_{A\to A^{c}}\left({\phi^{\left(-\right)}}_{A^{c}\to A}\left(g_{A^{c}}\right)\right)-g_{A^{c}}\right\|}_{2}<\delta$ **then**
   $\phi_{A^{c}\to A}\left(g_{A^{c}}\right)\leftarrow {\phi^{\left(-\right)}}_{A^{c}\to A}\left(g_{A^{c}}\right)$
  **end if**
 **end while**

**end for**

### Sensitivity of Average Explanations to Registration

The fidelity of the group‐level average deformation maps depends on the accuracy of the underlying image‐registration pipeline. As outlined in Vector Field Transformation, these average maps are generated by applying sampling and vector field registration operations to individual deformation fields based on the image registration fields. While the sampling and vector field operations maintain bounded errors, any instabilities within the individual deformation fields produced by the registration network could propagate and lead to misleading group‐level representations.

To evaluate the sensitivity of the average deformation maps to registration uncertainty, we introduced varying magnitudes of random, voxel‐wise noise to the output registration fields prior to the aggregation steps described in Vector Field Transformation. The resulting Jacobian determinants of the average deformations for $\text{Classifie}r_{\mathrm{PD}}$ are illustrated in Figure A4. As expected, escalating noise thresholds led to a proportional increase in background deformation magnitude, confirming that the averaging pipeline faithfully reflects the underlying registration inputs. Despite this, the structural stability of the average explanations remains evident, as the core anatomical features from the noise‐free baseline are robustly preserved when voxel‐wise noise levels are kept below 1%, as shown in Figure A4.
